# Comparison of Novel Systemic Inflammatory Indices for Identification of Left Ventricular Hypertrophy in Hypertensive Patients: A Large-Scale Cross-Sectional Study in China

**DOI:** 10.3390/jcdd13090457

**Published:** 2026-09-10

**Authors:** Ning Yang, Qing Zhu, Menghui Wang, Wenbo Yang, Jing Hong, Shasha Liu, Sharezhati Yishajiang, Mengru Wang, Nanfang Li

**Affiliations:** 1 Graduate School, Xinjiang Medical University, Urumqi 830054, China; 2 Hypertension Center of People’s Hospital of Xinjiang Uygur Autonomous Region, Xinjiang Hypertension Institute, NHC Key Laboratory of Hypertension Clinical Research, Key Laboratory of Xinjiang Uygur Autonomous Region “Hypertension Research Laboratory”, Xinjiang Clinical Medical Research Center for Hypertension (Cardio-Cerebrovascular) Diseases, People’s Hospital of Xinjiang Uygur Autonomous Region, NO.91 TianChi Road, Urumqi 830001, Chinayangwenbo8@126.com (W.Y.); xjhypertension@aliyun.com (J.H.);

**Keywords:** left ventricular hypertrophy, inflammatory indices, hypertension, diagnostic accuracy

## Abstract

**Background:** Inflammation plays a key role in the onset of hypertension and left ventricular hypertrophy (LVH); large-scale studies on inflammatory markers in hypertension-related LVH are lacking. This study aims to investigate the relationship between inflammatory markers and LVH in hypertensive patients and evaluate their clinical diagnostic value. **Methods:** The present cross-sectional study included 21,486 patients diagnosed with hypertension from a China Hospital. Six inflammatory indices are calculated based on complete blood counts, including the aggregate index of systemic inflammation (AISI), Systemic Inflammation Response Index (SIRI), Systemic Immune Inflammation Index (SII), Neutrophil-to-Lymphocyte Ratio (NLR), Neutrophil-to-Platelet Ratio (NPR) and the Platelet-to-Lymphocyte Ratio (PLR). Multivariate logistic regression models, receiver operating characteristic (ROC) curves, the area under the curve (AUC), the net reclassification improvement (NRI), the improved discriminant index (IDI), decision curve analysis (DCA), sensitivity analysis and subgroup analysis were performed. **Results:** Levels of all inflammatory indices were significantly higher in hypertensive patients with LVH compared to those without LVH. Following multivariate adjustment, the odds of prevalent LVH increased by 76%, 48% and 39% for each one-standard-deviation increase in SII, NLR and AISI, respectively. After incorporating SII into the traditional risk factor model, the AUC of the combined diagnostic model increased significantly to 0.750, with an NRI of 0.437 and an IDI of 0.047. The AUC values for the combined models incorporating NLR, AISI, SIRI, PLR and NPR were 0.736, 0.728, 0.726, 0.720 and 0.719, respectively. **Conclusions:** In hypertensive patients, inflammatory indices, particularly SII, demonstrate a significant association with LVH and may provide incremental value for risk stratification beyond traditional risk factors. Further studies are needed to evaluate the clinical efficacy of these indices in management LVH.

## 1. Introduction

Hypertension is a significant modifiable risk factor for cardiovascular disease and mortality globally. Recent epidemiological data indicate that over 1.4 billion individuals worldwide are afflicted with hypertension, although the control rate continues to be alarming. Ref. [1] left ventricular hypertrophy (LVH) is a significant manifestation of target organ damage resulting from hypertension. It is not only a compensatory remodeling in response to long-term increased hemodynamic load, but also a key indicator of irreversible pathological progression in ventricular remodeling. Research has shown that, regardless of blood pressure management, people with hypertension and concurrent LVH face a markedly elevated risk of severe cardiovascular incidents, such as heart failure, malignant arrhythmias, and sudden cardiac death [2,3]. Consequently, the early identification of LVH and risk stratification in hypertensive patients, followed by targeted interventions, are of critical importance for optimizing clinical management and improving long-term patient outcomes.

The significance of chronic low-grade inflammation and dysregulated immune system activation in the onset and advancement of hypertension and cardiac remodeling is increasingly acknowledged [4,5]. It has been demonstrated by preceding studies that sustained immune–inflammatory responses have the capacity to expedite hypertension-related target organ damage by means of promoting vascular endothelial dysfunction, oxidative stress, neurohumoral activation, and fibrogenic responses [6,7]. Inflammatory cells and their released cytokines contribute to vascular remodeling and are pivotal in myocardial hypertrophy and interstitial fibrosis, ultimately resulting in morphological and functional problems in the left ventricle [4]. Peripheral blood cells are considered to be pivotal mediators in establishing a link between systemic inflammatory states and cardiovascular remodeling. Among these, neutrophils can mediate tissue damage and amplify inflammation by releasing reactive oxygen species (ROS) and proteases; lymphocytes reflect the body’s immune regulatory capacity and stress status; and platelet activation can further promote microvascular inflammation, endothelial damage, and a procoagulant state [8,9]. These inflammatory and immune responses interact to drive the progression of myocardial hypertrophy and fibrosis in hypertensive patients. At present, the diagnosis of LVH in clinical practice is primarily reliant upon echocardiography. However, the performance and interpretation of echocardiography are contingent on the examiner’s experience, and access to this technology is limited in primary care settings. Consequently, identifying cost-effective, convenient, and reliable biomarkers in peripheral blood that reflect systemic inflammatory burden holds significant clinical value for the early detection of LVH in hypertensive patients.

Composite inflammation indices derived from blood cell counts have garnered substantial interest in recent years for their ability to indicate systemic inflammatory burden and have shown considerable promise in the early detection and risk stratification of cardiovascular illnesses [10]. Previous studies have also demonstrated the significant clinical potential of the Neutrophil-to-Lymphocyte Ratio (NLR), the Neutrophil-to-Platelet Ratio (NPR) and the Platelet-to-Lymphocyte Ratio (PLR) for risk stratification and early intervention in cardiovascular events [11,12,13,14]. NPR may represent a composite marker reflecting systemic inflammatory status and immune–thrombotic interactions, which may be involved in the development of hypertensive cardiac remodeling. Additionally, innovative composite inflammatory markers, such as the Systemic Immune Inflammation Index (SII), Systemic Inflammation Response Index (SIRI), and Aggravated Inflammation System Index (AISI), are progressively being integrated into cardiovascular risk evaluation as they amalgamate data from diverse immune and inflammatory cells, thus offering a more comprehensive representation of imbalances in the body’s immune–inflammatory homeostasis [15,16,17,18]. Nonetheless, existing research on the correlation between inflammatory indicators and LVH related to hypertension is still insufficient. Previous research has predominantly concentrated on individual inflammatory markers and has utilized comparatively limited sample sizes, lacking comprehensive evaluations of several composite inflammatory markers in extensive real-world populations. The additional significance of these inflammatory markers in forecasting the likelihood of LVH remains inadequately assessed.

The present study systematically investigated the association between six hematological inflammatory indices (AISI, SIRI, SII, NLR, NPR, and PLR) and the risk of hypertensive LVH, and further examined their discriminative performance and risk reclassification value for LVH. The objective of the present study was to evaluate the diagnostic accuracy and potential influencing factors, thereby providing new clinical insights for the early identification and intervention in high-risk populations.

## 2. Materials and Methods

### 2.1. Study Population

**Figure 1:**
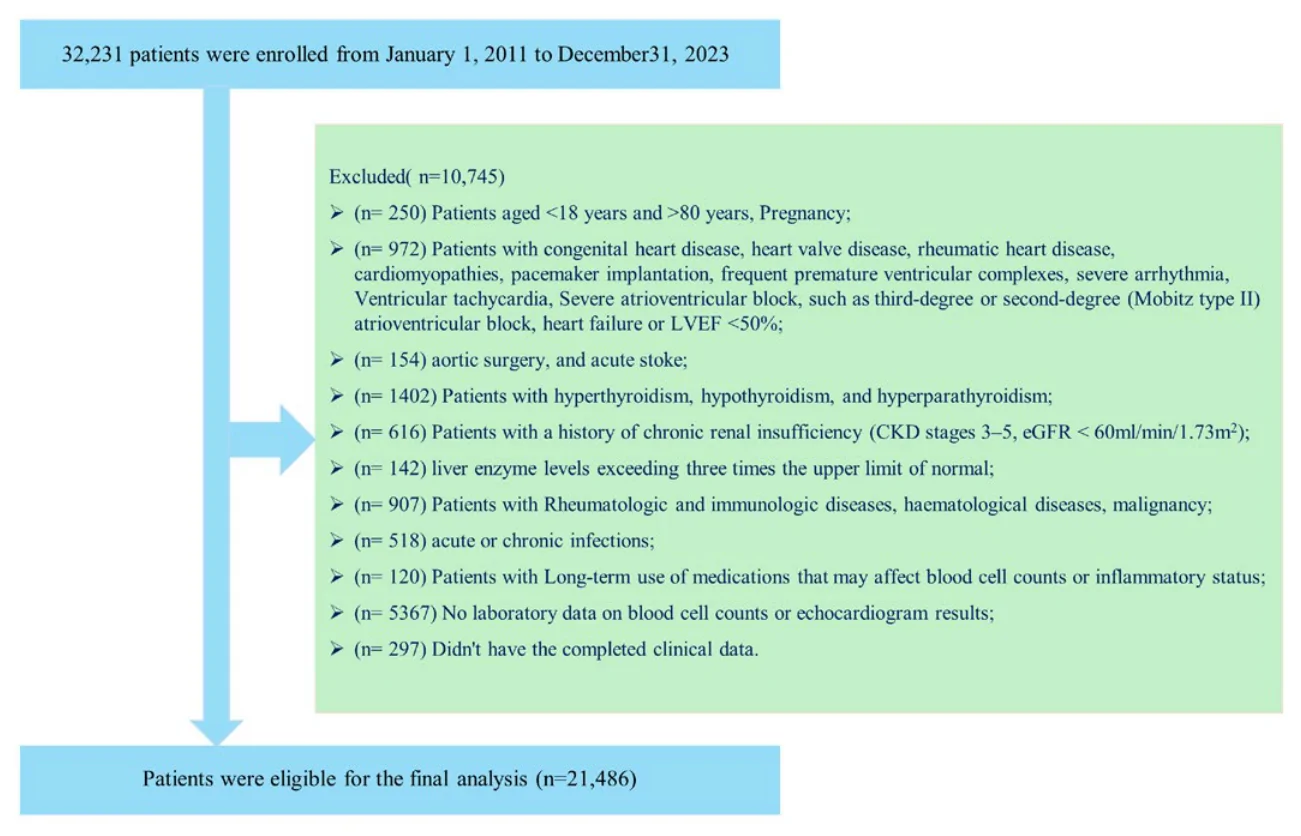
The flowchart of our study.

This exploratory investigation was structured as a cross-sectional study, with data gathered from 1 January 2011 to 31 December 2023 [19]. The present study initially included patients who had been hospitalized at the Hypertension Centre of the Xinjiang Uygur Autonomous Region People’s Hospital; all patients underwent standard complete blood count tests and echocardiography. To ensure the precision of LVH evaluation and to mitigate potential confounding biases, we instituted the following exclusion criteria: (1) age < 18 years or >80 years; (2) missing echocardiographic data or incomplete routine hematology test results; (3) pregnancy; (4) presence of conditions that may independently affect myocardial structure or inflammatory status, including cardiomyopathy, pacemaker implantation, congenital heart disease, heart valve disease, rheumatic heart disease, frequent premature ventricular complexes, severe arrhythmia, Ventricular tachycardia, Severe atrioventricular block, such as third-degree or second-degree (Mobitz type II) atrioventricular block, heart failure or left ventricular ejection fraction (LVEF) < 50%, and aortic surgery. (5) Stroke; (6) hyperthyroidism, hypothyroidism, parathyroid disease, chronic kidney disease (Stages 3, 4, 5), or eGFR < 60 mL/min/1.73 m^2^; (7) rheumatologic and immunologic diseases, hematological diseases, malignancies, liver enzyme levels exceeding three times the upper limit of normal, and acute or chronic infections; (8) prolonged administration of medicines that may influence hematologic markers or inflammatory conditions. Subsequent to the application of these criteria, a total of 21,486 participants were incorporated into the analysis (Figure 1). The research obtained ethical approval from the Ethics Committee of the People’s Hospital of the Xinjiang Uygur Autonomous Region (KY2024120158) and adheres to the ethical standards established in the Declaration of Helsinki and its subsequent revisions.

### 2.2. Data Collection and Definitions

We retrieved extensive data from the electronic medical record system, including clinical data such as demographic characteristics, physical examination results, lifestyle factors, and medication regimens. The data collected at admission encompassed demographic information such as age and sex, as well as anthropometric data including height, weight, body mass index (BMI), and blood pressure. The usage of smoking and alcohol was classified as either “current” or “non-current”. For a comprehensive account of the methodologies, comorbidities encompassed in the study, and definitions of additional variables, please consult the Supplementary Materials. Peripheral venous blood samples were collected from the individuals following an 8 to 10 h overnight fast to assess blood cell counts and biochemical markers. We collected the following biochemical parameters: fasting blood glucose (FBG), glycated hemoglobin (HbA1c), total cholesterol (TC), triglycerides (TG), high-density lipoprotein cholesterol (HDL-C), low-density lipoprotein cholesterol (LDL-C), alanine aminotransferase (ALT), aspartate aminotransferase (AST), serum creatinine (Scr), uric acid (UA), serum potassium, serum sodium, and high-sensitivity C-reactive protein (hs-CRP). The triglyceride-glucose (TyG) index is determined using the following formula: TyG = ln ([TG (mg/dL) × FBG (mg/dL)]/2) [20]. Complete blood counts, encompassing white blood cells, neutrophils, monocytes, lymphocytes, and platelets, were assessed utilizing a fully automated hematology analyzer(XN-3000; Sysmex Corporation, Kobe, Japan). The inflammatory indices calculated are listed below: AISI, SIRI, SII, NLR, NPR, and PLR. The following formulae were utilized in the calculation of the indices:

− AISI = Neutrophil count × Platelet count × (Monocyte count/Lymphocyte count);
− SIRI = Neutrophil count × (Monocyte count/Lymphocyte count);
− SII = Platelet count × Neutrophil count/Lymphocyte count;
− NLR = Neutrophil count/Lymphocyte count;
− NPR = Neutrophil count/Platelet count;
− PLR = Platelet count/Lymphocyte count.

### 2.3. Echocardiographic Characterization and Assessment of LVH

The echocardiographic measurements were conducted in accordance with the recommendations of the American Society of Echocardiography. Two researchers cross-checked the echocardiographic data in order to verify the accuracy of the recorded values and the completeness of the required variables. Echocardiography was used to assess the following parameters: left ventricular end-diastolic diameter (LVEDD), left ventricular end-systolic diameter (LVESD), left ventricular posterior wall thickness (LVPWT), interventricular septum thickness (IVST), left ventricular end-diastolic volume (LVEDV), LVEF. The present study utilized the 2.7th power of height to calculate the left ventricular mass index (LVMI = LVM/height^2.7^), given that the hypertensive patients included in this study exhibited a relatively high overall BMI [21]. The formula used to calculate LVMI was as follows: LVMI (g/m^2.7^) = (1.04 × [LVEDD + IVST + LVPWT]^3^ − LVEDD^3^ − 13.6)/height^2.7^ [21,22]. LVH was characterized by an LVMI of ≥50 g/m^2.7^ in males and ≥47 g/m^2.7^ in females [21]. The relative wall thickness (RWT) was determined utilizing the following formula: (2 × LVPWT)/LVEDD. The left ventricular geometry was classified into the following categories: concentric hypertrophy (RWT > 0.42), eccentric hypertrophy (RWT ≤ 0.42), concentric remodeling (normal LVMI with increased RWT), and normal (normal LVMI and normal RWT) [21,23].

### 2.4. Statistical Analysis

In this study, firstly, the missForest method was employed in R to impute missing data, then the participants were grouped by LVH status, and subsequently, the main analysis was conducted using the imputed dataset (Table S1). Initial multicollinearity analysis found VIF < 10 for all candidate variables, though TC had relatively high VIF in multivariate models for each inflammatory marker (Figure S1, Table S2). Due to the overlap between TC and LDL-C in biological composition and clinical information, we retained LDL-C and excluded TC from the final model to reduce lipid-related redundancy and model instability. We have also included a correlation matrix to provide a comprehensive illustration of the degree of correlation among the six inflammatory indices (Figure S2). A multivariable logistic regression model was employed to analyze the independent relationships between each inflammatory biomarker and the prevalence of LVH. The odds ratio (OR) and its 95% confidence interval (CI) were then calculated to measure the strength and accuracy of these connections. Restricted cubic splines (RCSs) were employed to model the dose–response relationship between inflammatory markers and the prevalence of LVH. We conducted sensitivity analyses to verify the robustness of the main results and performed stratified analyses to identify potential factors that may modulate the aforementioned associations. We evaluated the diagnostic performance of each model utilizing ROC curves and calculated the area under the curve (AUC), along with sensitivity, specificity, positive predictive value (PPV), negative predictive value (NPV), net reclassification improvement index (NRI) and integrated discrimination improvement index (IDI). Additionally, we employed DCA to evaluate the clinical net benefit of each inflammatory measure across several risk thresholds. All statistical analyses were conducted using R software (version 4.3.2), with a significance threshold of *p* < 0.05 for two-sided testing. Detailed information on the statistical methods can be found in the Supplementary Materials.

## 3. Results

### 3.1. Characteristics of the Study Population

The study sample comprised 10,291 hypertension patients without LVH and 11,195 hypertensive patients with LVH; their baseline characteristics are outlined in Table 1.

**Figure 2:**
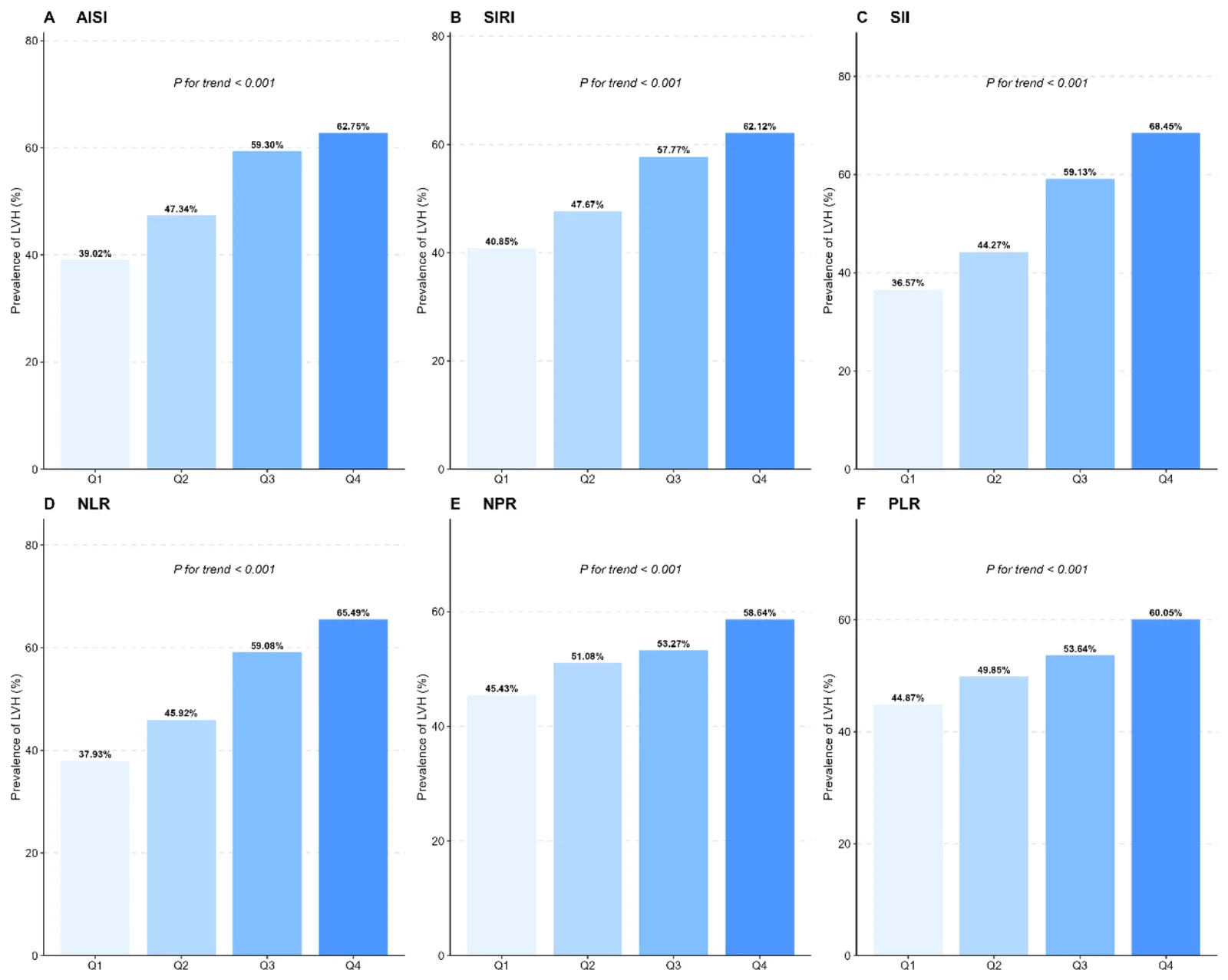
Relationship of inflammatory biomarkers with the prevalence of LVH, stratified by quartiles. (**A**) AISI; (**B**) SIRI; (**C**) SII; (**D**) NLR; (**E**) NPR; (**F**) PLR.

Compared with the non-LVH group, patients in the LVH group were older (51.96 ± 11.32 years vs. 47.92 ± 11.52 years, *p* < 0.001) and had a higher proportion of females (49.02% vs. 36.19%, *p* < 0.001). The BMI was markedly elevated in the LVH group (*p* < 0.001). Blood pressure assessments indicated substantial disparities, with elevated systolic and diastolic values noted in the LVH group (*p* < 0.001). Biochemical indicators revealed substantial disparities in FBG, HbA1c levels, and hs-CRP, with elevated values noted in the LVH group (all *p* < 0.001). In comparison to non-LVH patients, the duration of hypertension was prolonged, and there was a higher prevalence of comorbidities such as diabetes mellitus (DM) and coronary artery disease (CAD) in the LVH group (all *p* < 0.001). Medication utilization was significantly higher in the LVH group compared to non-LVH patients (all *p* < 0.001). The inflammatory indices (AISI, SIRI, SII, NLR, NPR, and PLR) were markedly increased in the LVH group. LVMI, LVM, LVEDD, LVDSD, LVPWT, IVST, and RWT were all markedly elevated in the LVH group compared to the non-LVH group, although LVEF was diminished. Figure 2 shows a dose–response relationship, with the prevalence of LVH, stratified by quartiles, increasing progressively with rising levels of each inflammatory marker. The prevalence of all six indices increased monotonically from Q1 to Q4, and the odds of having LVH rose progressively between each adjacent quartile group for each inflammatory marker (all trend tests: *p* < 0.001).

### 3.2. Relationship Between Inflammatory Indices and the Prevalence of LVH

We employed a multivariable logistic regression model to systematically assess the relationship between six inflammatory indicators (AISI, SIRI, SII, NLR, NPR, and PLR) and the prevalence of LVH, with results presented in Table 2. The research revealed that all six inflammatory indices were significantly and positively associated with the odds of having LVH. In the fully adjusted model, a one- standard deviation (SD) increase in SII was associated with a 76% increase in the odds of having LVH, with an OR of 1.76 (95% CI: 1.70–1.82, *p* < 0.001), representing the strongest effect among all markers. NLR and AISI also showed significant associations: for each one-SD increase, the odds of having LVH increased by 48% and 39%, respectively (NLR: OR = 1.48, 95% CI: 1.43–1.53; AISI: OR = 1.39, 95% CI: 1.35–1.44; both *p* < 0.001). SIRI, PLR and NPR demonstrated weaker but still significant positive associations: for each one-SD increase, the odds of having LVH increased by 35%, 24% and 18%, respectively (SIRI: OR = 1.35, 95% CI: 1.30–1.39; PLR: OR = 1.24, 95% CI: 1.20–1.28; NPR: OR = 1.18, 95% CI: 1.14–1.22; all *p* < 0.001). After stratifying each inflammatory marker by quartile (Q1–Q4), the odds of having LVH showed a clear dose–response trend. For example, compared with the lowest quartile (Q1), the odds of having LVH in the Q4 group were almost double (OR = 3.60, 95% CI: 3.30–3.94). AISI, SIRI, NLR, NPR and PLR exhibited a similar pattern, with OR values increasing progressively with each successive quartile, with the highest quartile showing the greatest risk (all trend test *p*-values < 0.001).

**Figure 3:**
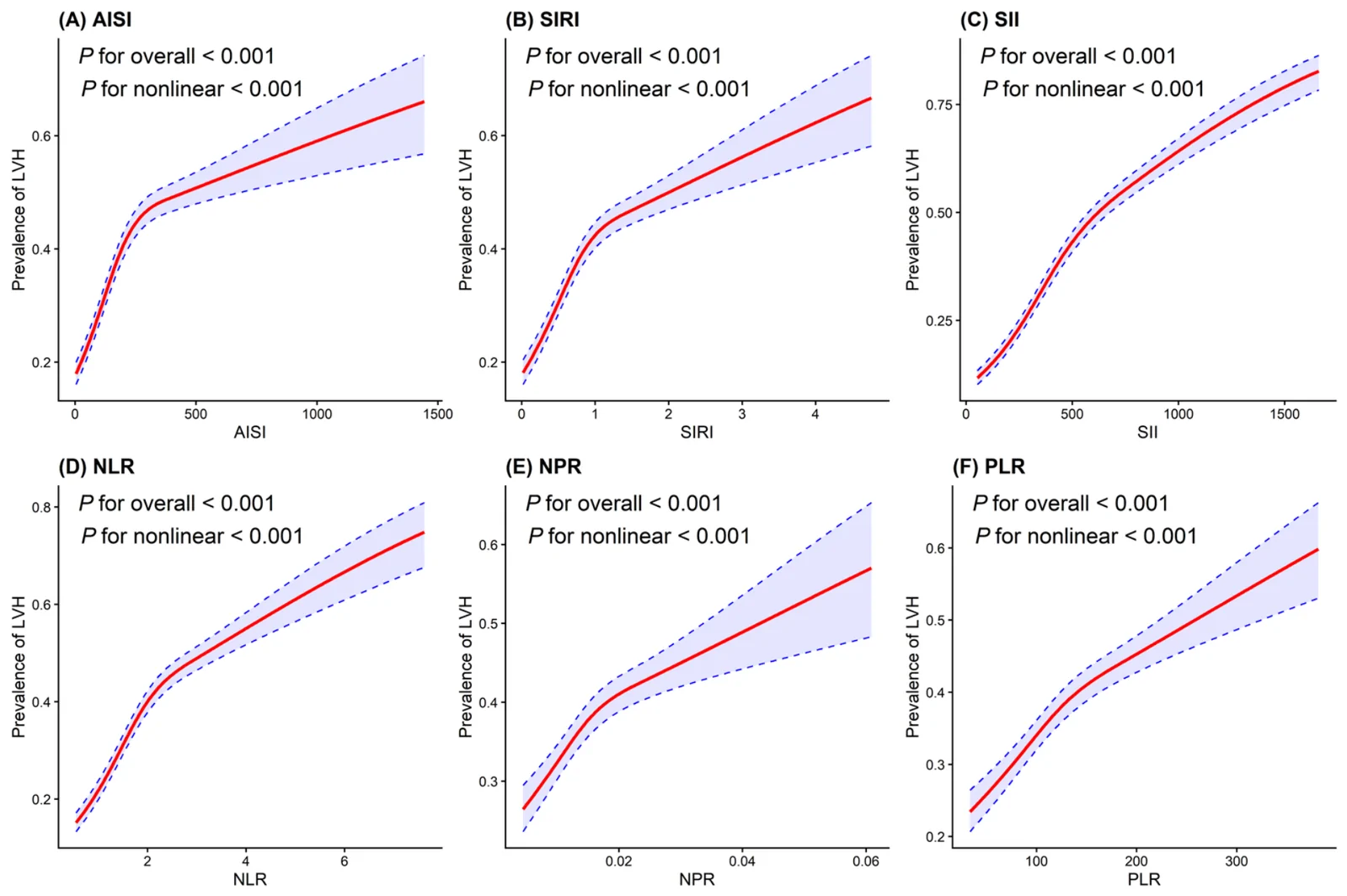
Dose–response relationship between inflammatory biomarkers and the prevalence of LVH. (**A**) AISI; (**B**) SIRI; (**C**) SII; (**D**) NLR; (**E**) NPR; (**F**) PLR.

RCS analysis was employed to elucidate the dose–response relationship between the composite inflammation index and the prevalence of LVH (Figure 3). The results demonstrated a significant, non-linear favorable relationship between all six inflammatory indicators and the odds of having LVH (*p* overall < 0.001 and *p* non-linear < 0.001).

### 3.3. Sensitivity Analysis

We conducted sensitivity analyses to assess the stability of the relationship between inflammatory indices and the prevalence of LVH. Table S3 shows that the results of the analysis after excluding outliers are consistent with those of the primary analysis, suggesting that the observed associations were not significantly altered by outliers. To control for the potential confounding effects of CAD, we excluded patients with CAD and reanalysed the data; the results remained consistent with those of the primary analysis, indicating that CAD did not substantially confound the association between inflammatory indices and LVH (Table S4). Furthermore, when patients with DM were excluded, the effect estimates for each inflammatory marker in the four models fluctuated slightly, but neither the direction nor the significance of the associations changed, suggesting that the association between inflammatory indices and the odds of having LVH is not influenced by DM status (Table S5: Sensitivity analysis of the association between inflammatory biomarkers and the odds of LVH without adjustment for Hs-CRP). The results demonstrated that the direction and statistical significance of the associations between each inflammatory marker and LVH remained consistent (Table S6). Sensitivity analyses conducted for different time periods showed that the ORs (95% CIs) for AISI were 1.93 (1.76–2.13), 1.36 (1.29–1.43) and 1.40 (1.32–1.48) for the periods 2011–2015, 2016–2019 and 2020–2023, respectively. The corresponding ORs (95% CIs) for SIRI were 1.71 (1.56–1.87), 1.31 (1.25–1.38) and 1.41 (1.33–1.49). In contrast, no clear period interaction was observed for SII, NLR, NPR and PLR, with *p*-values for interaction of 0.065, 0.863, 0.573 and 0.680, respectively (Table S7).

### 3.4. Subgroup Analysis

**Figure 4:**
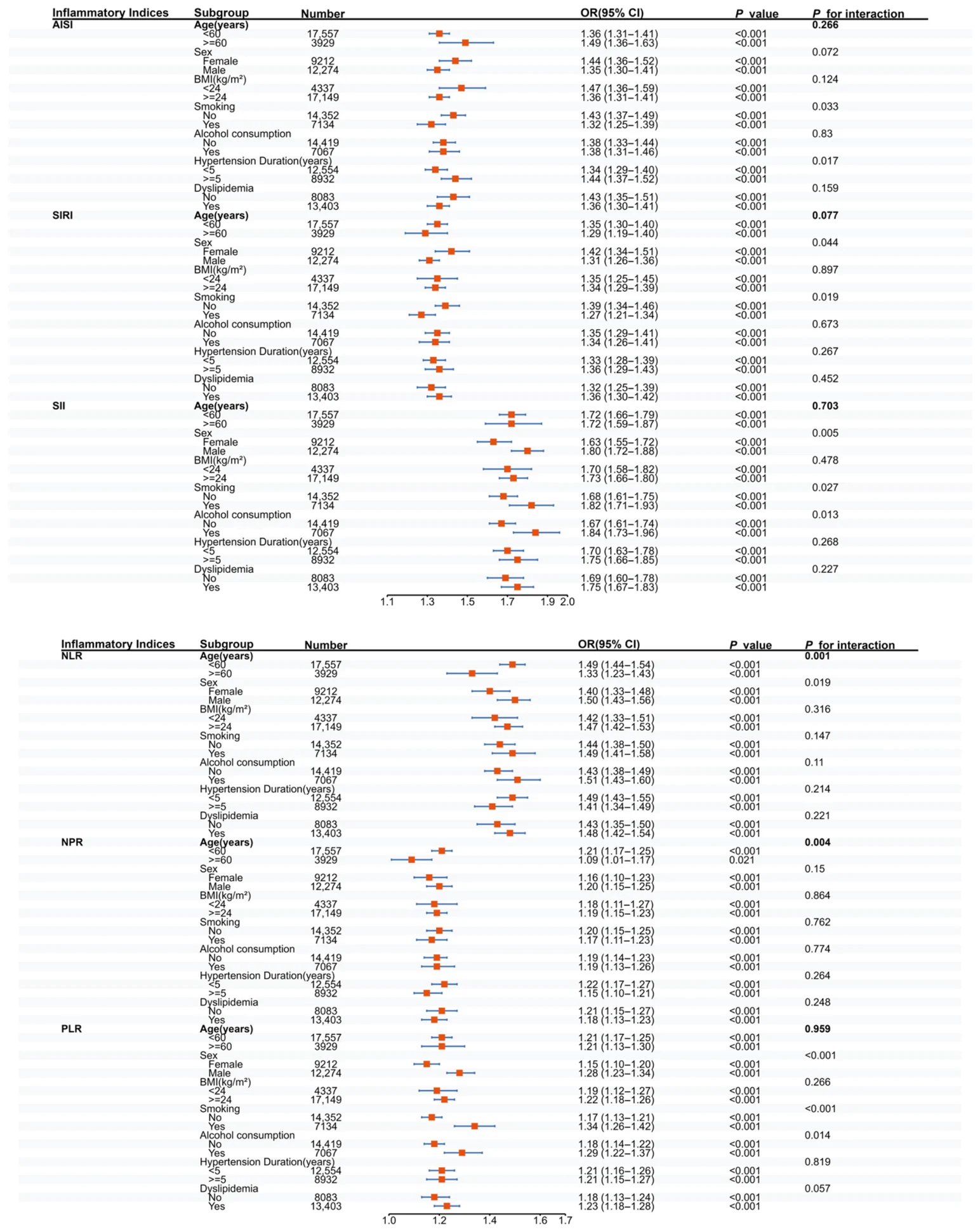
Stratified analysis of inflammatory biomarkers (per SD increasement) and their association with LVH.

Subgroup analyses were conducted according to age, sex, BMI, smoking status, alcohol intake, and the duration of hypertension and dyslipidemia (Figure 4, Table S8). Subgroup analyses demonstrated a strong positive relationship between all six inflammatory indices and the odds of having LVH across all categories, with a consistent directional effect among these markers. Interaction tests revealed that the *p*-values for interactions between certain subgroups reached statistical significance (e.g., the interaction between age and NLR, *p* = 0.001; the interaction between sex and SII, *p* = 0.005), suggesting that the magnitude of the effect may exhibit heterogeneity across different subgroups. However, the confidence intervals for the odds ratios overlapped across all subgroups and the direction of the effect was consistently positive with no instances of reversal. Therefore, these statistical interactions do not alter the core conclusion: there exists a universal positive association between the six inflammatory indices and the odds of having LVH. The subgroup analysis results affirm the efficacy of these inflammatory indicators in detecting the odds of having LVH across diverse demographic and clinical populations, indicating their broad beneficial applicability. We conducted a subgroup analysis stratified by drug class to assess the robustness of the association between inflammatory markers and LVH across different treatment settings. The results demonstrated that the direction of the association between inflammatory markers and LVH was consistent with that of the primary analysis across all drug-stratified subgroups, and no significant effect modification was observed (Figure S3).

### 3.5. Discriminative Performance and Clinical Utility of the Traditional Risk Factor and Inflammatory Indices Model for LVH

**Figure 5:**
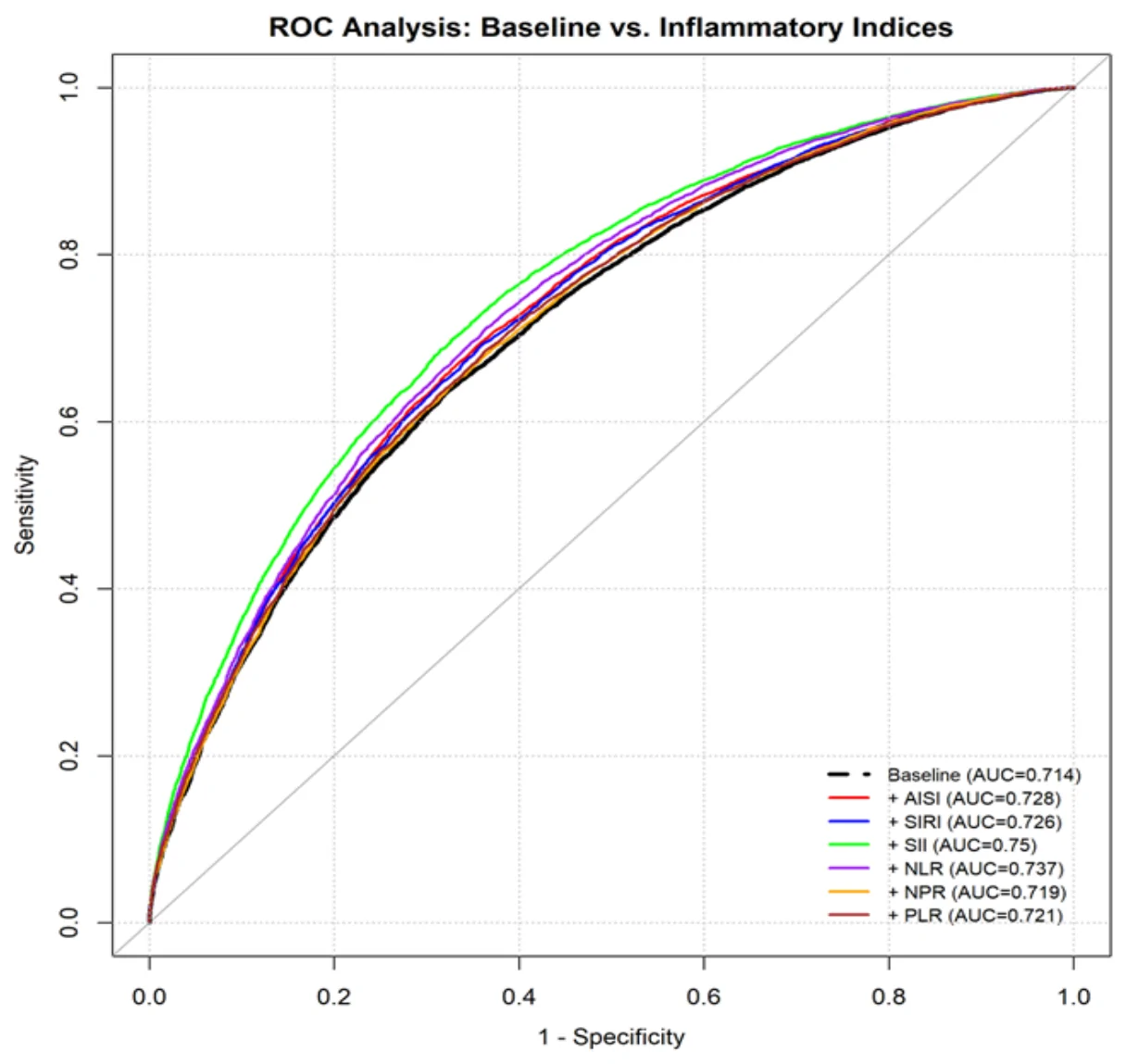
Receiver operating characteristic (ROC) curve analysis of combined model of inflammatory markers and traditional risk factors for LVH.

**Figure 6:**
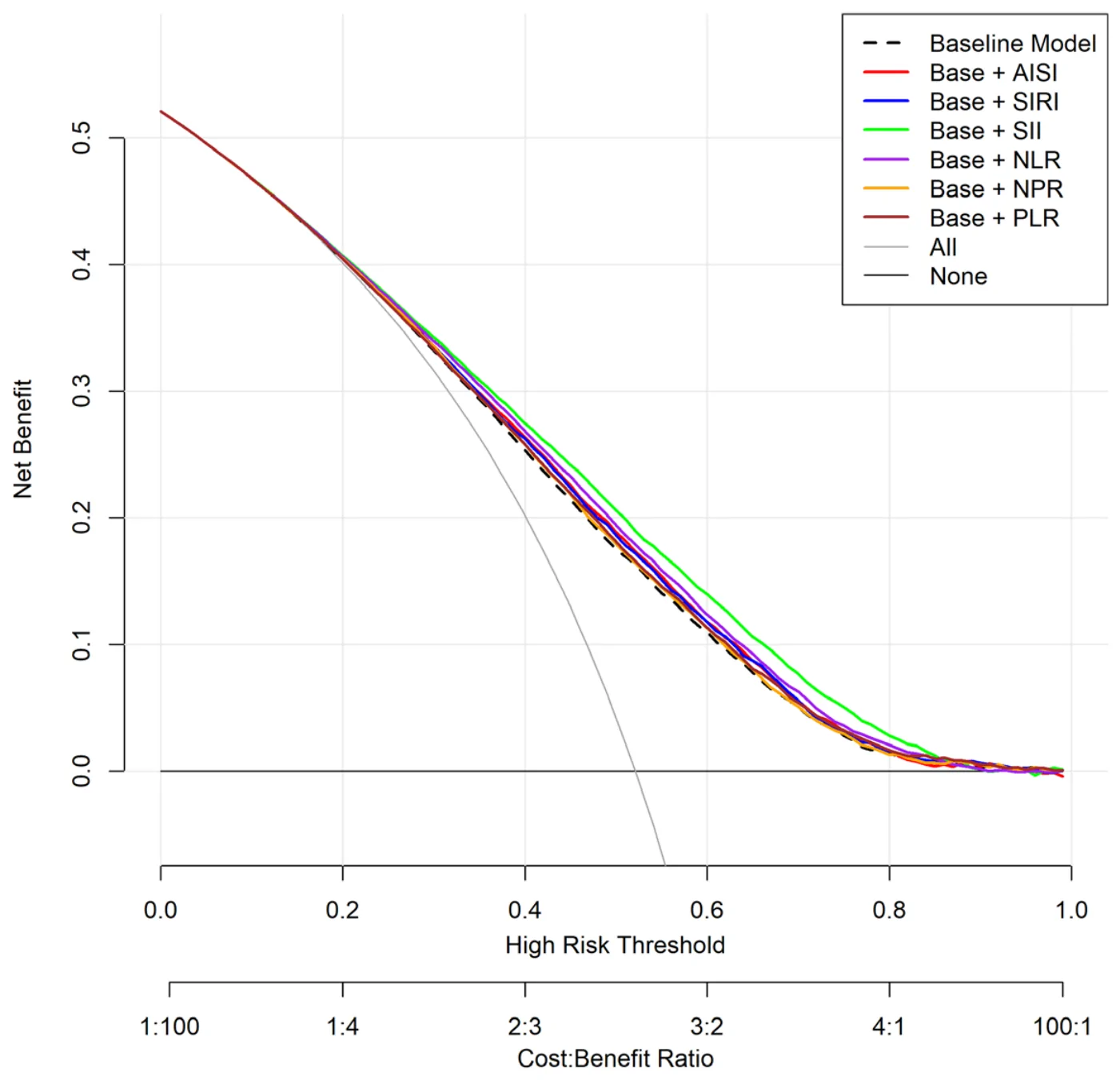
Decision curve analysis (DCA) of inflammatory markers and traditional risk factors for identification of LVH.

Six combined model was constructed by adding each inflammatory index individually to a traditional risk factor model (including age, sex, systolic blood pressure, BMI, DM, and smoking status). The model’s discriminative ability for LVH was assessed using ROC curves, NRI, IDI, and DCA (Figure 5 and Figure 6, Table 3 and Table 4). The AUC of the baseline model was 0.714; following the incorporation of SII, the AUC of the combined model increased significantly to 0.750 (ΔAUC = 0.036, DeLong test, *p* < 0.001), with a specificity of 0.644, a sensitivity of 0.728, a PPV of 0.690 and a NPV of 0.685. The NRI was 0.437 (*p* < 0.001) and the IDI was 0.047 (*p* < 0.001), indicating that SII significantly improved the ability to reclassify risk and the overall discriminatory power for LVH. The remaining inflammatory indices also demonstrated some incremental value: the AUC for Base + NLR was 0.736, with a NRI of 0.367 and an IDI of 0.028; for Base + AISI, the AUC was 0.728, with an NRI of 0.315 and an IDI of 0.017; for Base + SIRI, the AUC was 0.726, with an NRI of 0.292 and an IDI of 0.015; for Base + PLR, the AUC was 0.720, with an NRI of 0.200 and an IDI of 0.008; and for Base + NPR, the AUC was 0.719, with an NRI of 0.146 and an IDI of 0.006, with all *p*-values <0.001. However, in comparison with SII, the enhancement in these metrics was modest, and the clinical value-added was limited.

DCA further validated these findings from a clinical utility perspective (Figure 6). Throughout a broad spectrum of threshold probabilities, the Base + SII integrated model produced a higher net benefit than alternative combined models, including the baseline model.

We assessed the diagnostic accuracy of six inflammatory indices when applied individually for detecting LVH (Figures S4 and S5, Table S9). Among these, SII demonstrated the greatest discriminatory ability, with an AUC of 0.656 (95% CI: 0.649–0.663), a specificity of 0.620, a sensitivity of 0.617, a PPV of 0.639 and a NPV of 0.598. Overall, the discriminatory ability of individual inflammatory indices ranged from poor to moderate (AUC < 0.70). DCA further indicated that, within clinically relevant threshold probability ranges, the net benefit of individual indices was significantly lower than that of the combined model. These findings suggest that testing for SII in addition to traditional risk factors can improve the diagnostic accuracy of LVH and clinical decision-making efficiency without significantly increasing the cost of intervention. We employed the Bootstrap resampling method (with 1000 resamples) to perform internal validation and optimistic correction on all joint models (Figure S6, Table S10). The adjusted calibration slopes for all models were close to 1.0 (0.9971–0.9987), with Brier scores ranging from 0.2029 to 0.2150, indicating that the models were well calibrated and had acceptable predictive accuracy.

## 4. Discussion

The present study involved a comprehensive analysis of 21,486 hypertensive individuals, revealing that six composite inflammatory indices (AISI, SIRI, SII, NLR, NPR, and PLR) were independently linked to an elevated odds of having LVH. Among these indices, SII exhibited the most significant association: in the fully adjusted model, a one-standard-deviation rise in SII was associated with a 76% elevation in the odds of having LVH (OR = 1.76, 95% CI: 1.70–1.82). RCS curves revealed significant non-linear dose–response relationships between each inflammatory marker and the prevalence of LVH (*p* overall < 0.001 and *p* non-linear < 0.001). A series of sensitivity analyses, including the elimination of outliers and the exclusion of individuals with CAD or DM, yielded results congruent with the primary findings, thus demonstrating the robustness of the study outcomes. Furthermore, the diagnostic model constructed by integrating SII with the traditional risk factor model demonstrated a significant enhancement in the AUC to 0.750 (ΔAUC = 0.036), accompanied by significant advancements in both the NRI and the IDI; DCA further validated that this amalgamated model provides the greatest clinical net benefit. The results of this study support the view that systemic inflammation plays a significant role in hypertension-related LVH. The SII serves as a straightforward and accessible indicator of inflammation, and it may provide incremental value for facilitating identification of LVH risk stratification beyond traditional risk factors in hypertensive patients, necessitating additional confirmation through prospective research. Despite the model including a large number of control variables, internal validation using the Bootstrap method revealed only a minimal decline in performance metrics, indicating an extremely low risk of overfitting; the conclusions of this study remain robust and reliable.

The pathophysiological mechanisms that underpin the association between inflammation and LVH encompass a multitude of immune activation processes, with immune system activation and chronic low-grade inflammation playing key driving roles [4,24]. In hypertensive states, mechanical stress, activation of the renin–angiotensin–aldosterone system (RAAS), and oxidative stress have been demonstrated to induce vascular endothelial damage and the recruitment of immune cells, thereby triggering a systemic low- grade inflammatory response [25]. Activated immune cells can migrate to target organs, including the heart, where they release pro-inflammatory cytokines such as IL-6 and TNF-α, thereby promoting the proliferation of cardiac fibroblasts, collagen deposition, and cardiac interstitial fibrosis, ultimately driving left ventricular remodeling [25]. Hypertension triggers left ventricular remodeling through sustained pressure overload, a process accompanied by a chronic low-grade inflammatory response where elevated blood pressure subjects blood vessels and cardiomyocytes to stretch stress, thereby activating a series of signaling pathways, including the release of pro-inflammatory cytokines such as TNF-α and interleukin-6 [26]. These inflammatory cytokines attract inflammatory cells, including neutrophils, monocytes, and macrophages, to infiltrate the myocardium; by releasing mediators such as ROS and matrix metalloproteinases (MMPs), they promote collagen fiber deposition and fibroblast proliferation, resulting in myocardial fibrosis and hypertrophy. Research by Mehta et al. has shown that LVH is linked to a persistent state of low-grade inflammation, indicating that inflammation may play a crucial role in promoting myocardial remodeling [26,27].

A systematic review investigated a relationship between SII and cardiovascular disease, revealing that elevated SII levels are significantly associated with the risk of cardiovascular disease and major adverse cardiovascular events (MACEs) in individuals with coronary heart disease [28]; another systematic review and meta-analysis corroborated that SII is highly effective in prognostic assessment for patients with heart failure, indicating its potential as a predictive biomarker for cardiovascular disease overall [29]. The SII reflects the equilibrium between inflammation and immune status, integrating the synergistic effects of three key cell types: neutrophils, platelets, and lymphocytes; its elevation suggests that inflammatory cells are relatively overactive or that immune regulatory function is impaired [17,24]. Neutrophils exacerbate myocardial endothelial damage by releasing ROS and neutrophil extracellular traps (NETs); platelets not only participate in thrombus formation but also act as pro-inflammatory cells by releasing transforming growth factor-β (TGF-β), which directly drives myocardial fibrosis; meanwhile, the relative decline in lymphocyte levels reflects an immune imbalance in the body under chronic stress [30]. This sustained activation of the inflammatory immune response ultimately leads to pathological remodeling of the left ventricle by inducing phenotypic transformation of cardiac fibroblasts and collagen deposition [30,31,32]. Orhan Karayiğit et al. [33] showed in a study involving 150 hypertensive patients that SII can independently predict the development of LVH. The results of our study demonstrate the superior discriminative performance of SII in detecting LVH and are highly consistent with these findings. The present study extends these findings to a substantial population, confirming the significant association between SII and LVH in hypertensive patients and demonstrating, through multivariate analysis, that SII possesses independent incremental potential value beyond traditional risk factors. Given that the vast majority of patients in this study had already received antihypertensive therapy prior to hospitalization, it was not possible to compare differences in inflammation between treated and untreated states. However, subgroup analyses, stratified by drug class, revealed that different treatment regimens did not significantly alter the association between inflammatory markers and LVH, suggesting that the findings of this study have good generalizability across different treatment contexts.

Moreover, the NLR has been evidenced in multiple investigations as a straightforward and dependable indicator of inflammation, exhibiting significant predictive significance in diverse cardiovascular conditions, such as hypertension, coronary heart disease, and heart failure [34,35]. Despite the evidence from numerous studies associating NLR with LVH [36,37], our study utilized a large-scale cross-sectional comparison to demonstrate that SII, which incorporates platelet information, yields superior ORs and higher diagnostic weights. This finding indicates that platelet-activated microvascular inflammation may have a substantial role in stress-induced cardiac hypertrophy. With regard to inflammatory indices such as AISI and SIRI, the current evidence base suggests that they are clinically valuable for cardiovascular risk stratification [38,39]. A recent study evaluated the roles of AISI and SIRI in atherosclerosis risk stratification, finding that SIRI demonstrated robust and consistent predictive ability for atherosclerosis risk across different populations, whereas the predictive performance of AISI was found to be population-dependent [40]. Our research identified a significant association between both AISI and SIRI and the odds of having LVH; importantly, this is the inaugural documented association between AISI and the odds of having LVH. Nevertheless, with regard to the predictive value and discriminatory ability of the indices, AISI and SIRI proved to be inferior to SII. Research on the association between PLR and LVH is limited [33], and no previous studies have reported on the relationship between NPR and the odds of having LVH. The present study is the first to report a positive association between NPR and the odds of having LVH, and furthermore, it was found that the effects of both PLR and NPR on LVH are relatively weak. The present study has significance for the clinical management of hypertensive patients in several critical areas. Firstly, as the optimal inflammatory marker in the combined model, SII significantly enhances the discriminative ability of traditional risk models for LVH. Secondly, DCA demonstrates that the combined Base + SII model produces a higher net benefit across various threshold probabilities; SII has the potential to provide initial identification of LVH for patients with hypertension, offering insights for implementing early, aggressive blood pressure-lowering strategies. Furthermore, a quartile-based analysis of the six inflammatory indices revealed a consistent dose–response trend, suggesting that the odds of having LVH increase progressively as the inflammatory burden accumulates.

The current research offers a comprehensive examination of the relationship between inflammation and LVH in patients with hypertension. The study’s considerable strength lies in its large sample size, which significantly bolsters statistical power for the systematic assessment of the relationship between several inflammatory indicators and LVH. Furthermore, by comparing six inflammatory indices, the study provides direct comparative evidence to aid in the clinical selection of the most appropriate inflammatory biomarker. The methodological approaches employed to assess clinical utility comprised the following: the RCS model for evaluating dose–response relationships; ROC analysis; and reclassification indices (NRI/IDI) for assessing discriminative value; and DCA for evaluating clinical net benefit. Despite SII demonstrating the strongest association with LVH among the inflammatory markers evaluated, its predictive ability when used alone remains limited (AUC = 0.656), suggesting that inflammatory markers alone are insufficient for detecting LVH. The potential clinical value may lie more in providing additional information when integrated into multifactorial clinical models. Although inflammatory markers enhanced the model’s discriminatory ability, overall discriminatory performance remained at a low to moderate level. Consequently, these markers are more suitable as auxiliary indicators than as independent diagnostic tools. Additionally, sensitivity and subgroup analyses were conducted to validate the robustness and generalizability of the findings. As hs-CRP and the inflammatory markers studied may overlap in some inflammatory pathways, adjusting for hs-CRP could lead to over-adjustment; however, sensitivity analyses conducted without adjusting for hs-CRP support the robustness of the main findings. All six inflammatory markers exhibited a consistent positive association with LVH across different time periods. However, the associations for AISI and SIRI were stronger in early-stage patients, while no significant temporal heterogeneity was observed for SII, NLR, NPR and PLR. These results suggest that the overall direction of the association is relatively stable, but changes in clinical management, laboratory testing and echocardiographic procedures over the long-term study period may have impacted the effect estimates for certain inflammatory markers. Subgroup analyses suggest that the association between certain inflammatory markers and LVH may be modified by age and sex. However, the direction of the association remained consistent across all subgroups, with no reversal of effect, suggesting that these interactions primarily reflect differences in the strength of association rather than fundamentally different patterns. Given the large sample size of this study and the numerous subgroup comparisons conducted, statistically significant interactions do not necessarily represent clinically significant heterogeneity; therefore, these exploratory findings require further validation in independent cohorts. In the present study, owing to the substantial sample size and high statistical power, the emphasis was placed on effect sizes and their clinical significance when interpreting differences between groups rather than on statistical significance alone. Although some traditional risk factors reached statistical significance, their clinical significance should be interpreted with caution due to their small effect sizes. The present study also has several limitations that require careful consideration. Firstly, the cross-sectional study design is unable to establish a causal relationship between inflammatory indices and LVH. Secondly, the study population consisted primarily of Chinese patients diagnosed with hypertension; given that it was based on a single-center cohort of hypertensive patients, selection bias may be present, and the findings still need to be validated in other populations. Thirdly, although a multitude potential confounding factors were taken into account in the analysis, certain elements (such as physical activity, lifestyle, and genetics) were not thoroughly incorporated and may have impacted the results. This study spans a long period of time, during which clinical management strategies, echocardiography techniques, and other factors may have undergone change; these time-related factors may have had some impact on the identification of LVH and the assessment of related indicators. Fourth, we used laboratory-based inflammatory markers to indirectly reflect inflammatory status rather than measuring specific inflammatory mediators such as cytokines.0 There is a certain degree of correlation among the biological information represented by inflammatory markers; therefore, comparisons between these markers should be interpreted with caution. Therefore, the findings of this study require further confirmation through prospective cohort studies and validation in independent external cohorts.

## 5. Conclusions

In conclusion, this study indicates that increased levels of six inflammatory indices are associated with the prevalence of LVH in hypertensive patients, with the SII emerging as the most promising inflammatory biomarker. The integration of SII with traditional risk factors resulted in a substantial enhancement of the discriminative capacity for LVH. These finding underscores the potential clinical significance of inflammatory indices in the identification of LVH in hypertensive patients. Further prospective studies and multicentre validation are required to provide greater clarity on the clinical utility of inflammatory indices in the management of LVH in hypertensive patients.

## Figures and Tables

**Table 1:** The clinical characteristics of LVH and non-LVH groups.

Characteristics	Non LVH (N = 10,291)	LVH (N = 11,195)	*p* Value	SMD/Cramer’s V
Age, years	47.92 ± 11.52	51.96 ± 11.32	<0.001	0.353
Female	3724 (36.19)	5488 (49.02)	<0.001	0.13
BMI, kg/m^2^	26.08 ± 3.46	28.04 ± 4.01	<0.001	0.52
Current smoking	3765 (36.59)	3369 (30.09)	<0.001	0.069
Current alcohol consumption	3785 (36.78)	3282 (29.32)	<0.001	0.079
Hypertension duration, n (%)			<0.001	0.132
<5 years	6710 (65.20)	5844 (52.20)		
≥5 years	3581 (34.80)	5351 (47.80)		
Diabetes Mellitus, n (%)	1303 (12.66)	2068 (18.47)	<0.001	0.08
CAD, n (%)	530 (5.15)	963 (8.60)	<0.001	0.068
Dyslipidemia, n (%)	6427 (62.45)	6976 (62.31)	0.833	0.001
SBP, mm Hg	143.72 ± 18.30	149.63 ± 20.96	<0.001	0.324
DBP, mm Hg	88.93 ± 13.31	90.84 ± 15.22	<0.001	0.133
FBG, mmol/L	4.91 ± 1.41	5.13 ± 1.65	<0.001	0.145
HbAlc	5.99 ± 1.00	6.06 ± 1.06	<0.001	0.076
TC, mmol/L	4.54 ± 0.98	4.52 ± 1.00	0.039	0.028
TG, mmol/L	1.89 ± 1.50	1.88 ± 1.45	0.492	0.009
LDL-C, mmol/L	2.77 ± 0.83	2.74 ± 0.85	0.005	0.038
HDL-C, mmol/L	1.06 ± 0.27	1.06 ± 0.26	0.408	0.011
ALT, U/L	28.82 ± 24.62	27.72 ± 29.90	0.003	0.04
AST, U/L	21.48 ± 13.05	21.48 ± 15.94	0.984	0
Scr, μmol/L	66.42 ± 13.92	64.40 ± 14.75	<0.001	0.141
eGFR, mL/min/1.73 m^2^	104.52 ± 16.06	100.58 ± 15.48	<0.001	0.25
UA, μmol/L	348.42 ± 94.58	339.96 ± 93.80	<0.001	0.09
Serum potassium, mmol/L	3.84 ± 0.34	3.79 ± 0.37	<0.001	0.141
Serum sodium, mmol/L	140.92 ± 2.46	141.09 ± 2.56	<0.001	0.069
Hs-CRP	2.62 ± 4.20	3.42 ± 6.42	<0.001	0.146
TyG index	8.71 ± 0.63	8.75 ± 0.62	<0.001	0.068
AISI	195.17 ± 134.05	239.63 ± 151.93	<0.001	0.31
SIRI	0.81 ± 0.47	0.95 ± 0.53	<0.001	0.277
SII	428.47 ± 195.94	543.51 ± 240.36	<0.001	0.522
NLR	1.87 ± 0.72	2.16 ± 0.81	<0.001	0.378
NPR	0.01 (0.01–0.02)	0.02 (0.01–0.02)	<0.001	0.17
PLR	126.52 ± 42.78	135.51 ± 45.10	<0.001	0.204
ACEI/ARB, n (%)	4371 (42.47)	5701 (50.92)	<0.001	0.085
β-blocker, n (%)	1586 (15.41)	2197 (19.62)	<0.001	0.055
CCB, n (%)	5440 (52.86)	7176 (64.10)	<0.001	0.114
Diuretic, n (%)	1203 (11.69)	1958 (17.49)	<0.001	0.082
lipid-lowering drugs, n (%)	880 (8.74)	1321 (11.90)	<0.001	0.054
Antidiabetic agents	590 (5.73)	918 (8.20)	<0.001	0.061
LVMI, g/m^2.7^	41.49 ± 5.54	57.56 ± 8.15	<0.001	2.288
LVM, g	175.90 ± 33.89	227.47 ± 41.42	<0.001	1.357
LVEDD, mm	45.48 ± 3.28	48.52 ± 3.16	<0.001	0.944
LVESD, mm	25.21 ± 3.31	26.56 ± 3.73	<0.001	0.383
LVPWT, mm	9.60 ± 0.89	10.56 ± 0.98	<0.001	1.016
IVST, mm	9.92 ± 0.99	11.02 ± 1.07	<0.001	1.058
LVEDV, mL	71.29 ± 14.85	76.59 ± 16.04	<0.001	0.342
LVEF, %	61.41 ± 4.13	60.55 ± 4.29	<0.001	0.204
RWT	0.42 ± 0.05	0.44 ± 0.05	<0.001	0.262
LV Geometric Patterns				
Concentric hypertrophy, n (%)	-	7063 (63.09)	-	
Eccentric hypertrophy, n (%)	-	4132 (36.91)	-	
Concentric remodeling, n (%)	5039 (48.97)	-	-	

Data are mean (standard deviation), n (%), or median (interquartile range). SBP, systolic blood pressure; DBP, diastolic blood pressure; BMI, body mass index; FBG, fasting blood glucose; HbA1c, glycosylated hemoglobin; TC, total cholesterol; TG, triglycerides; LDL-C, low density lipoprotein-cholesterol; HDL-C, high density lipoprotein-cholesterol; ALT, alanine aminotransferase; AST, aspartate aminotransferase; Scr, serum creatinine; eGFR, estimated glomerular filtration rate; UA, uric acid; hs-CRP, high sensitivity C-reactive protein; AISI, aggregate index of systemic inflammation; SIRI, systemic inflammatory response index; SII, Systemic Immune Inflammation Index; NLR, Neutrophil-to-Lymphocyte Ratio; NPR, Neutrophil-to-Platelet Ratio; PLR, Platelet-to-Lymphocyte Ratio; ACEI, angiotensin-converting enzyme inhibitor; ARB, angiotensin receptor blocker; CCB, calcium channel blocker. LVMI, left ventricular mass index; LVM, left ventricular mass; LVEDD, left ventricular end-diastolic diameter; LVESD, left ventricular end-systolic diameter; LVPWT, end-diastolic left ventricular posterior wall thickness; IVST, end-diastolic interventricular septum thickness; LVEDV, left ventricular end-diastolic volume; LVEF, left ventricular ejection fraction; RWT, Relative wall thickness; LVH, left ventricular hypertrophy.

**Table 2:** Multiple Logistic regression analysis of the association between inflammatory biomarkers and the odds of LVH.

	Unadjusted		Model 1		Model 2		Model 3	
	OR (95%CI)	*p* Value	OR (95%CI)	*p* Value	OR (95%CI)	*p* Value	or (95%ci)	*p* Value
AISI per SD increase	1.40 (1.36–1.45)	<0.001	1.40 (1.36–1.45)	<0.001	1.40 (1.36–1.45)	<0.001	1.39 (1.35–1.44)	<0.001
AISI quartiles								
Q1 (≤121.71)	Reference		Reference		Reference		Reference	
Q2 (121.71–187.12)	1.40 (1.30–1.52)	<0.001	1.38 (1.27–1.50)	<0.001	1.38 (1.27–1.50)	<0.001	1.37 (1.26–1.49)	<0.001
Q3 (187.12–274.14)	2.28 (2.11–2.46)	<0.001	2.27 (2.09–2.46)	<0.001	2.25 (2.06–2.44)	<0.001	2.22 (2.04–2.42)	<0.001
Q4 (>274.14)	2.63 (2.43–2.84)	<0.001	2.69 (2.47–2.92)	<0.001	2.65 (2.43–2.89)	<0.001	2.60 (2.38–2.84)	<0.001
*p* for trend		<0.001		<0.001		<0.001		<0.001
SIRI per SD increase	1.35 (1.31–1.39)	<0.001	1.38 (1.34–1.43)	<0.001	1.36 (1.31–1.40)	<0.001	1.35 (1.30–1.39)	<0.001
SIRI quartiles								
Q1 (≤0.54)	Reference		Reference		Reference		Reference	
Q2 (0.54–0.79)	1.32 (1.22–1.42)	<0.001	1.33 (1.23–1.45)	<0.001	1.30 (1.19–1.41)	<0.001	1.29 (1.19–1.40)	<0.001
Q3 (0.79–1.10)	1.98 (1.84–2.14)	<0.001	2.07 (1.90–2.25)	<0.001	2.01 (1.84–2.18)	<0.001	1.98 (1.82–2.16)	<0.001
Q4 (>1.10)	2.38 (2.20–2.57)	<0.001	2.57 (2.36–2.80)	<0.001	2.42 (2.22–2.64)	<0.001	2.39 (2.18–2.60)	<0.001
*p* for trend		<0.001		<0.001		<0.001		<0.001
SII per SD increase	1.78 (1.73–1.84)	<0.001	1.78 (1.72–1.84)	<0.001	1.77 (1.71–1.83)	<0.001	1.76 (1.70–1.82)	<0.001
SII quartiles								
Q1 (≤332.35)	Reference		Reference		Reference		Reference	
Q2 (332.35–445.82)	1.38 (1.28–1.49)	<0.001	1.37 (1.26–1.49)	<0.001	1.36 (1.25–1.48)	<0.001	1.35 (1.24–1.47)	<0.001
Q3 (445.82–593.03)	2.48 (2.33–2.72)	<0.001	2.49 (2.29–2.70)	<0.001	2.48 (2.28–2.70)	<0.001	2.45 (2.25–2.67)	<0.001
Q4 (>593.03)	3.76 (3.47–4.08)	<0.001	3.75 (3.44–4.08)	<0.001	3.67 (3.36–4.01)	<0.001	3.60 (3.30–3.94)	<0.001
*p* for trend		<0.001		<0.001		<0.001		<0.001
NLR per SD increase	1.51 (1.46–1.55)	<0.001	1.54 (1.49–1.59)	<0.001	1.49 (1.44–1.54)	<0.001	1.48 (1.43–1.53)	<0.001
NLR quartiles								
Q1 (≤1.49)	Reference		Reference		Reference		Reference	
Q2 (1.49–1.89)	1.39 (1.29–1.50)	<0.001	1.40 (1.29–1.52)	<0.001	1.37 (1.26–1.49)	<0.001	1.36 (1.25–1.48)	<0.001
Q3 (1.89–2.40)	2.36 (2.19–2.56)	<0.001	2.44 (2.25–2.66)	<0.001	2.33 (2.14–2.54)	<0.001	2.30 (2.12–2.51)	<0.001
Q4 (>2.40)	3.10 (2.87–3.36)	<0.001	3.29 (3.02–3.58)	<0.001	3.03 (2.77–3.30)	<0.001	2.98 (2.73–3.25)	<0.001
*p* for trend		<0.001		<0.001		<0.001		<0.001
NPR per SD increase	1.19 (1.16–1.23)	<0.001	1.22 (1.18–1.26)	<0.001	1.18 (1.14–1.21)	<0.001	1.18 (1.14–1.22)	<0.001
NPR quartiles								
Q1 (≤0.011)	Reference		Reference		Reference		Reference	
Q2 (0.011–0.015)	1.25 (1.16–1.35)	<0.001	1.25 (1.16–1.36)	<0.001	1.23 (1.13–1.34)	<0.001	1.22 (1.13–1.33)	<0.001
Q3 (0.015–0.018)	1.37 (1.27–1.48)	<0.001	1.39 (1.28–1.51)	<0.001	1.31 (1.20–1.43)	<0.001	1.31 (1.20–1.42)	<0.001
Q4 (>0.018)	1.70 (1.58–1.84)	<0.001	1.82 (1.67–1.98)	<0.001	1.66 (1.52–1.81)	<0.001	1.66 (1.52–1.81)	<0.001
*p* for trend		<0.001		<0.001		<0.001		<0.001
PLR per SD increase	1.23 (1.20–1.27)	<0.001	1.26 (1.23–1.30)	<0.001	1.25 (1.21–1.29)	<0.001	1.24 (1.20–1.28)	<0.001
PLR quartiles								
Q1 (≤100.60)	Reference		Reference		Reference		Reference	
Q2 (100.60–124.48)	1.22 (1.13–1.32)	<0.001	1.23 (1.14–1.34)	<0.001	1.22 (1.12–1.32)	<0.001	1.21 (1.11–1.31)	<0.001
Q3 (124.48–154.79)	1.42 (1.32–1.53)	<0.001	1.46 (1.34–1.58)	<0.001	1.43 (1.31–1.55)	<0.001	1.41 (1.30–1.53)	<0.001
Q4 (>154.79)	1.85 (1.71–1.99)	<0.001	1.94 (1.79–2.11)	<0.001	1.90 (1.74–2.07)	<0.001	1.86 (1.71–2.03)	<0.001
*p* for trend		<0.001		<0.001		<0.001		<0.001

Model 1: adjusted for age, sex, smoking, alcohol consumption, hypertension duration, BMI, CAD, diabetes mellitus, dyslipidemia; Model 2: adjusted for variables in Model 1, SBP, DBP, FBG, HbAlc, ALT, AST, TC, TG, HDL, LDL, Scr, UA, serum potassium, serum sodium, hs-CRP, TyG index. Model 3: adjusted for variables in Model 2 plus ACEI/ARB, CCB, β-blocker, Diuretic, lipid-lowering drugs, and antidiabetic agents. Abbreviations: SD, standard deviation; OR, odds ratio; CI, confidence interval. Other abbreviations are as defined in Table 1.

**Table 3:** Diagnostic performance of combined model of inflammatory markers and traditional risk factors for LVH.

Index	AUC	95%CI_Low	95%CI_High	Specificity	Sensitivity	PPV	NPV
Baseline	0.714	0.707	0.721	0.662	0.651	0.677	0.636
Baseline + AISI	0.728	0.721	0.735	0.659	0.679	0.684	0.653
Baseline + SIRI	0.726	0.719	0.733	0.648	0.685	0.679	0.654
Baseline + SII	0.75	0.744	0.757	0.644	0.728	0.69	0.685
Baseline + NLR	0.736	0.73	0.743	0.685	0.662	0.696	0.651
Baseline + NPR	0.719	0.712	0.726	0.67	0.648	0.681	0.636
Baseline + PLR	0.72	0.713	0.727	0.61	0.711	0.664	0.659

Abbreviations: AUC, area under the curve; Positive-pv, positive predictive value; Negative-pv, negative predictive value. Other abbreviations are as defined in Table 1.

**Table 4:** Discrimination of each diagnostic model for using continuous NRI and IDI.

Index	Base_AUC	Comb_AUC	DeLong_P	NRI_Est(95%)	NRI_P	IDI_Est(95%)	IDI_P
AISI	0.714	0.728	<0.001	0.313(0.286–0.340)	<0.001	0.017(0.015–0.018)	<0.001
SIRI	0.714	0.726	<0.001	0.292(0.265–0.319)	<0.001	0.015(0.013–0.017)	<0.001
SII	0.714	0.75	<0.001	0.438(0.411–0.464)	<0.001	0.048(0.045–0.050)	<0.001
NLR	0.714	0.737	<0.001	0.373(0.346–0.399)	<0.001	0.028(0.026–0.031)	<0.001
NPR	0.714	0.719	<0.001	0.142(0.116–0.169)	<0.001	0.006(0.005–0.007)	<0.001
PLR	0.714	0.721	<0.001	0.204(0.177–0.230)	<0.001	0.008(0.007–0.009)	<0.001

Abbreviations: AUC, area under the curve; NRI, net reclassification improvement; IDI, integrated discrimination improvement. Other abbreviations are as defined in Table 1.

## Data Availability

All data supporting the findings are in the manuscript. More detailed information and raw data can be obtained from the corresponding author upon reasonable request.

## Supplementary Materials

The following supporting information can be downloaded at: https://www.mdpi.com/article/10.3390/jcdd13090457/s1, Figure S1. Collinearity diagnostics step; Figure S2. Spearman correlation matrix among the six inflammatory indices; Figure S3. Association of inflammatory biomarkers with LVH stratified by medication use; Figure S4. Receiver operating characteristic (ROC) curve analysis of individual inflammatory biomarkers for LVH; Figure S5. Decision curve analysis of individual inflammatory biomarkers for the identification of LVH; Figure S6. Calibration curves of the predictive models for LVH after bootstrap optimism correction; Table S1. The description of missing data; Table S2. Covariance diagnostics; Table S3. Sensitivity analysis of the relationship between inflammatory biomarkers and the Odds of LVH by excluding outliers; Table S4. Sensitivity analysis of the relationship between inflammatory biomarkers and the Odds of LVH by excluding CAD; Table S5. Sensitivity analysis of the relationship between inflammatory biomarkers and the Odds of LVH by excluding DM; Table S6. Sensitivity analysis of the association between inflammatory biomarkers and the Odds of LVH without adjustment for Hs-CRP; Table S7. Sensitivity analysis of the associations between inflammatory indices and LVH across different recruitment periods; Table S8. Interaction estimates for seven subgroups in the associations between inflammatory indices and LVH; Table S9. Diagnostic Performance of Individual Inflammatory Biomarkers for LVH; Table S10. Bootstrap optimism-corrected internal validation of the predictive models for LVH.
