# Supplementary material for: Comparison of Novel Systemic Inflammatory Indices for Identification of Left Ventricular Hypertrophy in Hypertensive Patients: A Large-Scale Cross-Sectional Study in China
Source: J Cardiovasc Dev Dis. 2026 Sep 10;13(9):457. doi: 10.3390/jcdd13090457 (PMC13607570; doi:10.3390/jcdd13090457)
Supplement: Supplementary file 1 [file jcdd-13-00457-s001.zip › jcdd-4404003-supplementary.pdf]

# Supplementary Material

*Supplementary Material*

## S1. Supplemental material and methods

### S1.1 Baseline examination

Anthropometric assessments were conducted by trained nursing staff. Height and weight measurements were obtained using standardized protocols, ensuring accuracy to 0.1 cm and 0.1 kg, respectively. Current smoking status was defined based on a history of smoking at least 100 cigarettes in one's lifetime and current smoking habits. Alcohol consumption was assessed through inquiries about the types of alcoholic beverages consumed, the frequency of weekly alcohol intake, and the typical quantity consumed per occasion. Individuals reporting weekly alcohol intake exceeding 140 g for men and 70 g for women were classified as having excessive alcohol consumption. Blood pressure measurements were obtained using a mercury sphygmomanometer following a period of at least 10 minutes of quiet rest. Multiple readings were taken, and the average values were recorded for systolic and diastolic blood pressure. All biochemical analyses were conducted on blood samples collected after an overnight fast.

### S1.2 Definition

Body mass index (BMI) was calculated as weight in kilograms divided by the square of the height in meters. Hypertension was diagnosed based on self-reported history of hypertension, current use of antihypertensive medications, or systolic blood pressure (SBP) of 140 mmHg or higher and/or diastolic blood pressure (DBP) of 90 mmHg or higher, recorded on at least three consecutive occasions. Diabetes mellitus (DM) was identified by fasting serum glucose levels of 7.0 mmol/L or higher, 2-hour serum glucose levels of 11.1 mmol/L or higher following an oral glucose tolerance test, or the current use of hypoglycemic agents or insulin. Dyslipidemia was determined by self-reported history of dyslipidemia and/or treatment with lipid-lowering medications. Coronary artery disease (CAD) was diagnosed based on coronary CT angiography (CTA) or coronary angiography, the patient's self-reported history of myocardial infarction, percutaneous coronary intervention, or coronary artery bypass grafting.

### S1.3 Details of the statistical analyses

In this study, firstly the missForest method was employed in R to impute missing data, then the participants were grouped by LVH status, and subsequently the main analysis was conducted using the imputed dataset (Table S1). In the initial multicollinearity analysis, the VIF values for all candidate variables were less than 10; however, total cholesterol (TC) exhibited a relatively high VIF in the multivariate

models corresponding to each inflammatory marker (see Figure S1 and Table S2). Given the significant overlap between TC and low-density lipoprotein cholesterol (LDL-C) in terms of biological composition and clinical information, and to reduce information redundancy and potential model instability among lipid-related variables, we ultimately retained LDL-C, which has more direct clinical implications, and excluded TC from the final multivariate model. In consideration of the distribution characteristics of the aforementioned inflammatory markers, Spearman's correlation analysis was employed to calculate the correlation coefficients between AISI, SIRI, SII, NLR, NPR, and PLR in all possible pairs. Clinical and demographic characteristics were summarized using appropriate statistical measures, including proportions for categorical variables, means with standard deviations (SDs) for normally distributed continuous variables, and medians with interquartile ranges (IQRs) for continuous variables exhibiting skewed distributions. To assess differences in characteristics across various patient subgroups, we employed the chi-square test for categorical variables. For continuous variables with normal distributions, one-way analysis of variance (ANOVA) was utilized, whereas the Kruskal–Wallis test was applied for those with skewed distributions. Subsequently, we employed multivariable logistic regression models to investigate the association between inflammatory biomarkers and the prevalence of Left Ventricular Hypertrophy (LVH). The models were structured as follows:

- Unadjusted (univariate analysis) to provide an initial assessment of the relationship between each biomarker and LVH.
- Model 1: Adjusted for age, sex, smoking status, alcohol consumption, hypertension duration, BMI, CAD, DM, and dyslipidemia.
- Model 2: Further adjusted for systolic blood pressure, diastolic blood pressure, fasting blood glucose, glycated hemoglobin (HbA1c), aspartate aminotransferase (AST), alanine aminotransferase (ALT), TC, triglyceride (TG), high-density lipoprotein cholesterol (HDL-C), LDL-C, serum creatinine (Scr), uric acid (UA), serum potassium, serum sodium, high-sensitivity C-reactive protein (hs-CRP), and triglyceride glucose (TyG) index.
- Model 3: Extended Model 2 by including the use of antihypertensive drugs, lipid-lowering agents, and antidiabetic medications to control for the potential effects of pharmacological interventions on the outcomes. For each model, we calculated the odds ratio (OR) and the corresponding 95% confidence interval (CI) to quantify the strength and precision of the associations. The p-value for trend was determined by treating quartiles of the inflammatory biomarkers as a continuous variable within each model. To further explore the dose–response relationship between the inflammatory biomarkers and the prevalence of LVH, we employed restricted cubic spline (RCS) models. This approach allowed us to assess the nonlinear relationship and identify potential threshold effects by focusing on the smoothed curve fitting.To ensure the robustness of our findings, we performed a series of sensitivity analyses to assess the stability of our results under different conditions. First, we conducted a sensitivity analysis to verify the stability of the observed association between inflammatory biomarkers and the prevalence of LVH; we conducted an analysis excluding outliers. Specifically, we removed participants whose inflammatory biomarker values were more than three SD from the mean (either less than -3SD or more than +3SD). This criterion was applied to minimize

the influence of extreme values on our analytical outcomes. Second, to assess the influence of CAD, we repeated the main analysis after excluding participants with a history of CAD at baseline. This step was taken to determine whether CAD could confound the relationship between inflammatory biomarkers and LVH. Third, to evaluate the impact of diabetes mellitus, we conducted the primary analysis again after excluding participants with evidence of diabetes mellitus at baseline. Fourthly, to assess the impact of adjusting for hs-CRP on the effect estimates, we also conducted a sensitivity analysis without adjustment for hs-CRP. Based on the study period, we divided the participants into three cohorts: 2011-2015, 2016-2019, and 2020-2023. Within each period, we used fully adjusted multivariate logistic regression models, identical to those in the primary analysis, to assess the association between LVH and a one-standard-deviation increase in AISI, SIRI, SII, NLR, NPR and PLR. All sensitivity analyses were conducted using the same statistical models and assumptions as the primary analysis to ensure consistency and comparability of results. In this stratified analysis, we aimed to identify potential effect modifiers of the association between inflammatory biomarkers and the prevalence of LVH. The analysis incorporated the following covariates: sex (male, female), age (<60 years, ≥60 years), current smoking status (yes, no), current alcohol consumption status (yes, no), hypertension duration (<5 years, ≥5 years), and presence of dyslipidemia (yes, no). To assess heterogeneity across subgroups, we employed logistic regression models. The results are graphically represented in forest plots, which facilitate the visualization of the effect estimates and their confidence intervals across different strata. Interactions between subgroups and inflammatory biomarkers were statistically evaluated using likelihood ratio tests, allowing us to determine whether the association between inflammatory biomarkers and LVH risk varied significantly across the subgroups defined by the covariates. The present study built upon a traditional risk factor model (including age, sex, systolic blood pressure, body mass index, history of diabetes, and smoking status) by incorporating six inflammatory biomarkers to construct six combined models. Given the mathematical overlap and potential correlation among the six inflammatory indices, each inflammatory index (AISI, SIRI, SII, NLR, NPR, and PLR) was entered individually into a separate multivariable model with the same set of covariates; the six indices were not included simultaneously in a single model. This approach was used to evaluate the association and incremental value of each inflammatory index under a consistent covariate-adjustment framework while minimizing potential multicollinearity arising from their shared blood cell components. The identification performance of this joint model for LVH was evaluated using receiver operating characteristic (ROC) curves, net reclassification improvement (NRI), integrated discriminant improvement (IDI) and decision curve analysis (DCA). In this study, we assessed the diagnostic accuracy of multiple biomarkers for the identification of LVH. We constructed ROC curves for the following biomarkers: the aggregate index of systemic inflammation (AISI), systemic inflammatory response index (SIRI), systemic immune inflammation index (SII), neutrophil-to-lymphocyte ratio (NLR), neutrophil-to-platelet ratio (NPR), and platelet-to-lymphocyte ratio (PLR). For each biomarker, we calculated the area under the ROC curve (AUC), which quantifies the test's discriminatory ability to distinguish between individuals with and without LVH. For each inflammatory index, the optimal cutoff value for

discriminating between patients with and without LVH was determined from the ROC curve by maximizing the Youden index (sensitivity+specificity-1). Sensitivity and specificity were subsequently calculated at the corresponding cutoff. The ROC-derived thresholds and their diagnostic performance were estimated using the same study population; no independent validation cohort was available. The incremental value of each inflammatory index beyond the base model was further evaluated using the continuous NRI and IDI. Continuous NRI was used throughout the analyses; therefore, no predefined risk categories were specified. Furthermore, we determined the sensitivities, specificities, positive predictive values, and negative predictive values to evaluate the identification of these indices for LVH. DCA was performed to evaluate the potential clinical utility of adding each inflammatory index to the base model across threshold probabilities ranging from 0% to 100%. The DCA curves, which plot threshold probabilities on the x-axis against standardized net benefits on the y-axis, were utilized to gauge the performance of the models. The net benefit of each model was compared with the treat-all and treat-none strategies. The threshold probability represents the relative weighting of false-positive and false-negative classifications in deciding whether further evaluation for LVH would be warranted. We employed the Bootstrap resampling method (with 1,000 resamples) to perform internal validation and optimistic correction on all joint models. The statistical analyses were performed using R software, version 4.3.2 (R Foundation for Statistical Computing), with statistical significance defined as a 2-sided P value less than 0.05.

## S2. Supplementary Tables

**Table S1.** The description of missing data.

| Variables | Total | Non-missing | Missing | Missing Ratio |
|---|---|---|---|---|
| Age, years | 21486 | 21486 | 0 | 0.00 |
| Female | 21486 | 21486 | 0 | 0.00 |
| BMI | 21486 | 21486 | 0 | 0.00 |
| Current smoking | 21486 | 21486 | 0 | 0.00 |
| Current alcohol consumption | 21486 | 21486 | 0 | 0.00 |
| Hypertension duration | 21486 | 21486 | 0 | 0.00 |
| Diabetes Mellitus | 21486 | 21486 | 0 | 0.00 |
| CAD | 21486 | 21486 | 0 | 0.00 |
| Dyslipidemia | 21486 | 21486 | 0 | 0.00 |
| SBP | 21486 | 21486 | 0 | 0.00 |
| DBP | 21486 | 21486 | 0 | 0.00 |
| FBG | 21486 | 21486 | 0 | 0.00 |
| HbAlc | 21486 | 18047 | 3439 | 0.16 |
| TC | 21486 | 21377 | 109 | 0.01 |
| TG | 21486 | 21376 | 110 | 0.01 |
| LDL-C | 21486 | 21375 | 111 | 0.01 |
| HDL-C | 21486 | 21390 | 96 | 0.00 |
| ALT | 21486 | 21387 | 99 | 0.00 |
| AST | 21486 | 21387 | 99 | 0.00 |
| Scr | 21486 | 21482 | 4 | 0.00 |
| UA | 21486 | 21482 | 4 | 0.00 |
| Serum potassium | 21486 | 21482 | 4 | 0.00 |
| Serum sodium | 21486 | 21482 | 4 | 0.00 |
| Hs-CRP | 21486 | 19034 | 2452 | 0.11 |
| ACEI/ARB | 21486 | 21486 | 0 | 0.00 |
| β-blocker | 21486 | 21486 | 0 | 0.00 |
| CCB | 21486 | 21486 | 0 | 0.00 |
| Diuretic | 21486 | 21486 | 0 | 0.00 |
| lipid-lowering drugs | 21486 | 21486 | 0 | 0.00 |
| Antidiabetic agents | 21486 | 21486 | 0 | 0.00 |
| Neutrophil | 21486 | 21486 | 0 | 0.00 |
| Monocyte count | 21486 | 21486 | 0 | 0.00 |
| Lymphocyte count | 21486 | 21486 | 0 | 0.00 |
| Platelet count | 21486 | 21486 | 0 | 0.00 |
| LVEDD | 21486 | 21486 | 0 | 0.00 |
| LVESD | 21486 | 20431 | 1055 | 0.05 |
| LVPWT | 21486 | 21486 | 0 | 0.00 |
| IVST | 21486 | 21486 | 0 | 0.00 |

| | | | | |
|---|---|---|---|---|
| LVEDV | 21486 | 21450 | 36 | 0.00 |
| LVEF | 21486 | 21466 | 20 | 0.00 |

Abbreviations are as defined in Table 1.

**Table S2.** Covariance diagnostics.

| Variable | AISI -VIF | SIRI -VIF | SII -VIF | NLR- VIF | PLR- VIF | NPR -VIF |
|---|---|---|---|---|---|---|
| AISI | 5.742 | - | - | - | - | - |
| SIRI | - | 7.133 | - | - | - | - |
| SII | - | - | 5.843 | - | - | - |
| NLR | - | - | - | 5.219 | - | - |
| PLR | - | - | - | - | 5.295 | - |
| NPR | - | - | - | - | - | 7.758 |
| Age | 1.7 | 1.702 | 1.707 | 1.702 | 1.698 | 1.697 |
| Sex | 2.893 | 2.887 | 2.884 | 2.891 | 2.884 | 2.882 |
| BMI | 1.281 | 1.283 | 1.272 | 1.277 | 1.275 | 1.277 |
| Hypertension duration | 1.244 | 1.244 | 1.243 | 1.243 | 1.243 | 1.243 |
| Current smoking | 1.933 | 1.934 | 1.934 | 1.936 | 1.931 | 1.93 |
| Current drinking | 1.869 | 1.863 | 1.865 | 1.864 | 1.864 | 1.863 |
| DM | 2.241 | 2.243 | 2.23 | 2.235 | 2.242 | 2.247 |
| CAD | 1.14 | 1.141 | 1.143 | 1.142 | 1.142 | 1.141 |
| Dyslipidemia | 1.483 | 1.483 | 1.489 | 1.482 | 1.481 | 1.48 |
| SBP | 2.044 | 2.045 | 2.039 | 2.047 | 2.049 | 2.05 |
| DBP | 2.457 | 2.46 | 2.449 | 2.459 | 2.461 | 2.461 |
| FBG | 2.488 | 2.488 | 2.488 | 2.492 | 2.487 | 2.501 |
| HbA1c | 1.158 | 1.157 | 1.158 | 1.157 | 1.157 | 1.159 |
| ALT | 3.883 | 4.002 | 4.144 | 3.912 | 4.207 | 3.93 |
| AST | 3.579 | 3.697 | 3.836 | 3.6 | 3.91 | 3.629 |
| Scr | 1.856 | 1.855 | 1.854 | 1.853 | 1.852 | 1.851 |
| UA | 1.72 | 1.719 | 1.711 | 1.717 | 1.716 | 1.719 |
| Total cholesterol | 9.719 | 9.751 | 9.687 | 9.787 | 9.786 | 9.798 |
| Triglyceride | 5.616 | 5.611 | 5.733 | 5.659 | 5.657 | 5.634 |
| HDL-C | 2.214 | 2.225 | 2.207 | 2.217 | 2.219 | 2.224 |
| LDL-C | 7.26 | 7.287 | 7.287 | 7.319 | 7.319 | 7.321 |
| hs-CRP | 1.102 | 1.108 | 1.103 | 1.108 | 1.106 | 1.107 |
| PLT | 2.009 | 1.306 | 2.97 | 1.303 | 3.812 | 4.609 |
| Lymphocyte count | 2.353 | 3.085 | 3.513 | 3.972 | 4.518 | 1.309 |
| Monocyte count | 3.041 | 4.256 | 1.453 | 1.459 | 1.459 | 1.459 |
| Neutrophils count | 2.751 | 3.712 | 4.227 | 5.008 | 1.412 | 7.169 |
| TyG index | 6.505 | 6.503 | 6.613 | 6.539 | 6.521 | 6.521 |
| ACEI/ARB | 1.266 | 1.266 | 1.268 | 1.265 | 1.265 | 1.265 |
| B-blockers | 1.116 | 1.117 | 1.117 | 1.117 | 1.117 | 1.116 |
| Diuretic | 1.176 | 1.177 | 1.176 | 1.177 | 1.176 | 1.176 |

| | | | | | | |
|---|---|---|---|---|---|---|
| CCB | 1.128 | 1.128 | 1.128 | 1.127 | 1.128 | 1.128 |
| Antidiabetic agents | 1.689 | 1.688 | 1.685 | 1.685 | 1.689 | 1.689 |
| Lipid-lowering drugs | 1.166 | 1.166 | 1.164 | 1.165 | 1.166 | 1.166 |

VIF = 1/(1-$R^2$). VIF step-by-step screening method: Calculate the VIF of each variable. Abbreviations: VIF, variance inflation factor. Other abbreviations are as defined in Table 1.

**Table S3.** Sensitivity analysis of the relationship between inflammatory biomarkers and the Odds of LVH by excluding outliers.

| | Unadjusted | | Model 1 | | Model 2 | | Model 3 | |
|---|---|---|---|---|---|---|---|---|
| | OR (95%CI) | *P* value | OR (95%CI) | *P* value | OR (95%CI) | *P* value | OR (95%CI) | *P* value |
| AISI per SD increase | 1.42(1.38-1.46) | <0.001 | 1.43(1.39-1.47) | <0.001 | 1.43(1.38-1.48) | <0.001 | 1.42(1.38-1.47) | <0.001 |
| AISI quartiles | | | | | | | | |
| Q1(≤120.64) | Reference | | Reference | | Reference | | Reference | |
| Q2(120.64-184.18) | 1.40(1.29-1.51) | <0.001 | 1.38(1.27-1.50) | <0.001 | 1.38(1.27-1.50) | <0.001 | 1.36(1.25-1.48) | <0.001 |
| Q3(184.18-268.45) | 2.23(2.07-2.42) | <0.001 | 2.23(2.05-2.42) | <0.001 | 2.21(2.03-2.41) | <0.001 | 2.19(2.01-2.39) | <0.001 |
| Q4(>268.45) | 2.64(2.44-2.86) | <0.001 | 2.69(2.47-2.93) | <0.001 | 2.67(2.45-2.92) | <0.001 | 2.63(2.41-2.87) | <0.001 |
| *P* for trend | | <0.001 | | <0.001 | | <0.001 | | <0.001 |
| SIRI per SD increase | 1.35(1.31-1.39) | <0.001 | 1.39(1.35-1.43) | <0.001 | 1.37(1.32-1.41) | <0.001 | 1.36(1.32-1.40) | <0.001 |
| SIRI quartiles | | | | | | | | |
| Q1(≤0.55) | Reference | | Reference | | Reference | | Reference | |
| Q2(0.55-0.79) | 1.29(1.20-1.39) | <0.001 | 1.31(1.20-1.42) | <0.001 | 1.28(1.17-1.39) | <0.001 | 1.27(1.16-1.38) | <0.001 |
| Q3(0.79-1.08) | 1.95(1.81-2.11) | <0.001 | 2.04(1.87-2.22) | <0.001 | 1.98(1.82-2.16) | <0.001 | 1.95(1.79-2.13) | <0.001 |
| Q4(>1.08) | 2.37(2.19-2.56) | <0.001 | 2.57(2.36-2.80) | <0.001 | 2.45(2.24-2.67) | <0.001 | 2.42(2.21-2.64) | <0.001 |
| *P* for trend | | <0.001 | | <0.001 | | <0.001 | | <0.001 |
| SII per SD increase | 1.75(1.70-1.80) | <0.001 | 1.75(1.69-1.80) | <0.001 | 1.74(1.69-1.80) | <0.001 | 1.73(1.67-1.79) | <0.001 |
| SII quartiles | | | | | | | | |
| Q1(≤329.00) | Reference | | Reference | | Reference | | Reference | |
| Q2(329.00-442.91) | 1.38(1.28-1.49) | <0.001 | 1.37(1.26-1.49) | <0.001 | 1.37(1.26-1.49) | <0.001 | 1.36(1.25-1.48) | <0.001 |
| Q3(442.91-585.70) | 2.45(2.27-2.65) | <0.001 | 2.43(2.23-2.64) | <0.001 | 2.42(2.23-2.64) | <0.001 | 2.40(2.20-2.61) | <0.001 |
| Q4(>585.70) | 3.78(3.49-4.09) | <0.001 | 3.77(3.46-4.10) | <0.001 | 3.71(3.40-4.06) | <0.001 | 3.65(3.34-3.99) | <0.001 |
| *P* for trend | | <0.001 | | <0.001 | | <0.001 | | <0.001 |
| NLR per SD increase | 1.52(1.48-1.56) | <0.001 | 1.56(1.51-1.61) | <0.001 | 1.51(1.47-1.56) | <0.001 | 1.50(1.46-1.55) | <0.001 |
| NLR quartiles | | | | | | | | |
| Q1(≤1.49) | Reference | | Reference | | Reference | | Reference | |

| | | | | | | | | |
|---|---|---|---|---|---|---|---|---|
| Q2(1.49-1.88) | 1.40(1.30-1.52) | <0.001 | 1.41(1.30-1.54) | <0.001 | 1.38(1.27-1.50) | <0.001 | 1.37(1.26-1.49) | <0.001 |
| Q3(1.88-2.38) | 2.34(2.17-2.53) | <0.001 | 2.42(2.22-2.63) | <0.001 | 2.30(2.12-2.51) | <0.001 | 2.28(2.09-2.48) | <0.001 |
| Q4(>2.38) | 3.11(2.88-3.37) | <0.001 | 3.29(3.02-3.58) | <0.001 | 3.05(2.80-3.33) | <0.001 | 3.01(2.76-3.28) | <0.001 |
| *P* for trend | | <0.001 | | <0.001 | | <0.001 | | <0.001 |
| NPR per SD increase | 1.19(1.16-1.22) | <0.001 | 1.22(1.19-1.26) | <0.001 | 1.18(1.15-1.22) | <0.001 | 1.18(1.15-1.22) | <0.001 |
| NPR quartiles | | | | | | | | |
| Q1(≤0.012) | Reference | | Reference | | Reference | | Reference | |
| Q2(0.012-0.015) | 1.25(1.16-1.35) | <0.001 | 1.25(1.15-1.36) | <0.001 | 1.23(1.13-1.34) | <0.001 | 1.22(1.13-1.33) | <0.001 |
| Q3(0.015-0.019) | 1.38(1.28-1.49) | <0.001 | 1.41(1.30-1.54) | <0.001 | 1.33(1.22-1.45) | <0.001 | 1.33(1.22-1.45) | <0.001 |
| Q4(>0.019) | 1.68(1.55-1.81) | <0.001 | 1.79(1.65-1.95) | <0.001 | 1.65(1.51-1.80) | <0.001 | 1.65(1.51-1.80) | <0.001 |
| *P* for trend | | <0.001 | | <0.001 | | <0.001 | | <0.001 |
| PLR per SD increase | 1.24(1.20-1.27) | <0.001 | 1.27(1.23-1.31) | <0.001 | 1.26(1.22-1.30) | <0.001 | 1.25(1.21-1.29) | <0.001 |
| PLR quartiles | | | | | | | | |
| Q1(≤100.40) | Reference | | Reference | | Reference | | Reference | |
| Q2(100.40-123.87) | 1.21(1.13-1.31) | <0.001 | 1.23(1.13-1.33) | <0.001 | 1.21(1.12-1.32) | <0.001 | 1.20(1.11-1.31) | <0.001 |
| Q3(123.87-153.46) | 1.40(1.30-1.52) | <0.001 | 1.45(1.33-1.57) | <0.001 | 1.42(1.30-1.54) | <0.001 | 1.40(1.29-1.52) | <0.001 |
| Q4(>153.46) | 1.85(1.71-1.99) | <0.001 | 1.94(1.78-2.11) | <0.001 | 1.89(1.74-2.07) | <0.001 | 1.86(1.70-2.03) | <0.001 |
| *P* for trend | | <0.001 | | <0.001 | | <0.001 | | <0.001 |

Values more than 3SD from the mean were deemed as outliers; they were removed and the analysis repeated.

Model 1: adjusted for age, sex, smoking, alcohol consumption, hypertension duration, BMI, CAD, diabetes mellitus, dyslipidemia;

Model 2: adjusted for variables in Model 1, SBP, DBP, FBG, HbAlc, ALT, AST, TC, TG, HDL, LDL, Scr, UA, serum potassium, serum sodium, hs-CRP, TyG index.

Model 3: adjusted for variables in Model 2 plus ACEI/ARB, CCB, β-blocker, diuretic, lipid-lowering agents, and antidiabetic agents.

Abbreviations: SD, standard deviation; OR, odds ratio; CI, confidence interval. Other abbreviations are as defined in Table 1.

**Table S4.** Sensitivity analysis of the relationship between inflammatory biomarkers and the Odds of LVH by excluding CAD.

| | Unadjusted | | Model 1 | | Model 2 | | Model 3 | |
|---|---|---|---|---|---|---|---|---|
| | OR (95%CI) | *P* value | OR (95%CI) | *P* value | OR (95%CI) | *P* value | OR (95%CI) | *P* value |
| AISI per SD increase | 1.40(1.36-1.45) | <0.001 | 1.40(1.36-1.45) | <0.001 | 1.41(1.36-1.46) | <0.001 | 1.40(1.35-1.45) | <0.001 |
| AISI quartiles | | | | | | | | |
| Q1(≤121.10) | Reference | | Reference | | Reference | | Reference | |
| Q2(121.10-185.80) | 1.42(1.31-1.53) | <0.001 | 1.40(1.28-1.52) | <0.001 | 1.40(1.28-1.52) | <0.001 | 1.38(1.27-1.51) | <0.001 |
| Q3(185.80-274.01) | 2.22(2.05-2.41) | <0.001 | 2.23(2.05-2.43) | <0.001 | 2.22(2.03-2.43) | <0.001 | 2.20(2.02-2.40) | <0.001 |
| Q4(>274.01) | 2.65(2.45-2.88) | <0.001 | 2.71(2.48-2.96) | <0.001 | 2.69(2.46-2.95) | <0.001 | 2.65(2.42-2.90) | <0.001 |
| *P* for trend | | <0.001 | | <0.001 | | <0.001 | | <0.001 |
| SIRI per SD increase | 1.35(1.31-1.39) | <0.001 | 1.39(1.34-1.43) | <0.001 | 1.37(1.32-1.41) | <0.001 | 1.36(1.31-1.41) | <0.001 |
| SIRI quartiles | | | | | | | | |
| Q1(≤0.54) | Reference | | Reference | | Reference | | Reference | |
| Q2(0.54-0.78) | 1.30(1.20-1.41) | <0.001 | 1.33(1.22-1.44) | <0.001 | 1.29(1.19-1.41) | <0.001 | 1.29(1.18-1.40) | <0.001 |
| Q3(0.78-1.10) | 1.98(1.83-2.14) | <0.001 | 2.08(1.91-2.26) | <0.001 | 2.01(1.85-2.20) | <0.001 | 2.00(1.83-2.18) | <0.001 |
| Q4(>1.10) | 2.40(2.21-2.60) | <0.001 | 2.61(2.39-2.85) | <0.001 | 2.47(2.26-2.70) | <0.001 | 2.44(2.23-2.67) | <0.001 |
| *P* for trend | | <0.001 | | <0.001 | | <0.001 | | <0.001 |
| SII per SD increase | 1.77(1.71-1.83) | <0.001 | 1.76(1.70-1.82) | <0.001 | 1.76(1.69-1.82) | <0.001 | 1.75(1.68-1.81) | <0.001 |
| SII quartiles | | | | | | | | |
| Q1(≤330.60) | Reference | | Reference | | Reference | | Reference | |
| Q2(330.60-445.00) | 1.36(1.25-1.47) | <0.001 | 1.35(1.24-1.47) | <0.001 | 1.34(1.23-1.47) | <0.001 | 1.33(1.22-1.45) | <0.001 |
| Q3(445.00-592.12) | 2.51(2.31-2.72) | <0.001 | 2.48(2.27-2.70) | <0.001 | 2.47(2.27-2.70) | <0.001 | 2.45(2.24-2.68) | <0.001 |
| Q4(>592.12) | 3.78(3.48-4.10) | <0.001 | 3.75(3.43-4.10) | <0.001 | 3.70(3.38-4.05) | <0.001 | 3.64(3.32-3.99) | <0.001 |
| *P* for trend | | <0.001 | | <0.001 | | <0.001 | | <0.001 |
| NLR per SD increase | 1.51(1.47-1.56) | <0.001 | 1.55(1.50-1.60) | <0.001 | 1.50(1.45-1.55) | <0.001 | 1.49(1.44-1.55) | <0.001 |
| NLR quartiles | | | | | | | | |
| Q1(≤1.49) | Reference | | Reference | | Reference | | Reference | |

| | | | | | | | | |
|---|---|---|---|---|---|---|---|---|
| Q2(1.49-1.88) | 1.41(1.30-1.53) | <0.001 | 1.42(1.31-1.55) | <0.001 | 1.39(1.28-1.52) | <0.001 | 1.38(1.27-1.51) | <0.001 |
| Q3(1.88-2.39) | 2.38(2.19-2.58) | <0.001 | 2.45(2.25-2.67) | <0.001 | 2.33(2.14-2.55) | <0.001 | 2.31(2.12-2.52) | <0.001 |
| Q4(>2.39) | 3.15(2.90-3.42) | <0.001 | 3.35(3.07-3.66) | <0.001 | 3.10(2.83-3.39) | <0.001 | 3.05(2.79-3.34) | <0.001 |
| *P* for trend | | <0.001 | | <0.001 | | <0.001 | | <0.001 |
| NPR per SD increase | 1.19(1.16-1.23) | <0.001 | 1.23(1.19-1.27) | <0.001 | 1.19(1.15-1.22) | <0.001 | 1.19(1.15-1.23) | <0.001 |
| NPR quartiles | | | | | | | | |
| Q1(≤0.011) | Reference | | Reference | | Reference | | Reference | |
| Q2(0.011-0.014) | 1.27(1.17-1.37) | <0.001 | 1.27(1.17-1.39) | <0.001 | 1.25(1.15-1.36) | <0.001 | 1.24(1.14-1.36) | <0.001 |
| Q3(0.014-0.018) | 1.39(1.28-1.50) | <0.001 | 1.43(1.31-1.56) | <0.001 | 1.35(1.24-1.47) | <0.001 | 1.34(1.23-1.47) | <0.001 |
| Q4(>0.018) | 1.69(1.57-1.83) | <0.001 | 1.84(1.68-2.01) | <0.001 | 1.68(1.53-1.84) | <0.001 | 1.68(1.53-1.84) | <0.001 |
| *P* for trend | | <0.001 | | <0.001 | | <0.001 | | <0.001 |
| PLR per SD increase | 1.23(1.20-1.27) | <0.001 | 1.26(1.22-1.30) | <0.001 | 1.25(1.21-1.29) | <0.001 | 1.24(1.20-1.28) | <0.001 |
| PLR quartiles | | | | | | | | |
| Q1(≤100.57) | Reference | | Reference | | Reference | | Reference | |
| Q2(100.57-124.48) | 1.22(1.13-1.32) | <0.001 | 1.23(1.13-1.34) | <0.001 | 1.22(1.12-1.33) | <0.001 | 1.21(1.11-1.32) | <0.001 |
| Q3(124.48-155.02) | 1.42(1.32-1.54) | <0.001 | 1.46(1.34-1.59) | <0.001 | 1.43(1.31-1.56) | <0.001 | 1.41(1.29-1.54) | <0.001 |
| Q4(>154.02) | 1.84(1.70-1.99) | <0.001 | 1.92(1.76-2.09) | <0.001 | 1.88(1.72-2.06) | <0.001 | 1.84(1.68-2.02) | <0.001 |
| *P* for trend | | <0.001 | | <0.001 | | <0.001 | | <0.001 |

Model 1: adjusted for age, sex, smoking, alcohol consumption, hypertension duration, BMI, diabetes mellitus, dyslipidemia;

Model 2: adjusted for variables in Model 1: SBP, DBP, FBG, HbAlc, ALT, AST, TC, TG, HDL, LDL, Scr, UA, serum potassium, serum sodium, hs-CRP, TyG index.

Model 3: adjusted for variables in Model 2 plus ACEI/ARB, CCB, β-blocker, Diuretic, lipid-lowering agents, and antidiabetic agents.

Abbreviations: SD, standard deviation; OR, odds ratio; CI, confidence interval. Other abbreviations are as defined in Table 1.

**Table S5.** Sensitivity analysis of the relationship between inflammatory biomarkers and the Odds of LVH by excluding DM.

| | Unadjusted | | Model 1 | | Model 2 | | Model 3 | |
|---|---|---|---|---|---|---|---|---|
| | OR (95%CI) | *P* value | OR (95%CI) | *P* value | OR (95%CI) | *P* value | OR (95%CI) | *P* value |
| AISI per SD increase | 1.40(1.35-1.44) | <0.001 | 1.41(1.36-1.46) | <0.001 | 1.41(1.36-1.46) | <0.001 | 1.40(1.35-1.45) | <0.001 |
| AISI quartiles | | | | | | | | |
| Q1(≤120.69) | Reference | | Reference | | Reference | | Reference | |
| Q2(120.69-185.08) | 1.39(1.28-1.51) | <0.001 | 1.39(1.27-1.52) | <0.001 | 1.39(1.27-1.52) | <0.001 | 1.37(1.25-1.51) | <0.001 |
| Q3(185.08-271.63) | 2.19(2.01-2.38) | <0.001 | 2.19(2.00-2.40) | <0.001 | 2.17(1.98-2.38) | <0.001 | 2.15(1.96-2.36) | <0.001 |
| Q4(>271.63) | 2.64(2.43-2.88) | <0.001 | 2.74(2.50-3.00) | <0.001 | 2.71(2.47-2.99) | <0.001 | 2.67(2.42-2.93) | <0.001 |
| *P* for trend | | <0.001 | | <0.001 | | <0.001 | | <0.001 |
| SIRI per SD increase | 1.34(1.30-1.38) | <0.001 | 1.39(1.34-1.43) | <0.001 | 1.36(1.32-1.41) | <0.001 | 1.36(1.31-1.41) | <0.001 |
| SIRI quartiles | | | | | | | | |
| Q1(≤0.54) | Reference | | Reference | | Reference | | Reference | |
| Q2(0.54-0.78) | 1.25(1.15-1.36) | <0.001 | 1.28(1.17-1.40) | <0.001 | 1.25(1.15-1.37) | <0.001 | 1.25(1.14-1.37) | <0.001 |
| Q3(0.78-1.09) | 1.84(1.70-2.00) | <0.001 | 1.95(1.78-2.13) | <0.001 | 1.90(1.73-2.08) | <0.001 | 1.87(1.71-2.06) | <0.001 |
| Q4(>1.09) | 2.36(2.17-2.57) | <0.001 | 2.60(2.37-2.85) | <0.001 | 2.46(2.24-2.71) | <0.001 | 2.43(2.21-2.67) | <0.001 |
| *P* for trend | | <0.001 | | <0.001 | | <0.001 | | <0.001 |
| SII per SD increase | 1.78(1.72-1.84) | <0.001 | 1.77(1.71-1.84) | <0.001 | 1.77(1.70-1.83) | <0.001 | 1.75(1.69-1.82) | <0.001 |
| SII quartiles | | | | | | | | |
| Q1(≤331.06) | Reference | | Reference | | Reference | | Reference | |
| Q2(331.06-444.45) | 1.39(1.27-1.51) | <0.001 | 1.37(1.25-1.50) | <0.001 | 1.36(1.24-1.50) | <0.001 | 1.35(1.23-1.48) | <0.001 |
| Q3(444.45-591.33) | 2.50(2.30-2.72) | <0.001 | 2.47(2.25-2.70) | <0.001 | 2.46(2.24-2.70) | <0.001 | 2.43(2.22-2.67) | <0.001 |
| Q4(>591.33) | 3.82(3.50-4.16) | <0.001 | 3.80(3.46-4.17) | <0.001 | 3.73(3.39-4.10) | <0.001 | 3.65(3.32-4.02) | <0.001 |
| *P* for trend | | <0.001 | | <0.001 | | <0.001 | | <0.001 |
| NLR per SD increase | 1.51(1.46-1.56) | <0.001 | 1.55(1.50-1.60) | <0.001 | 1.50(1.45-1.55) | <0.001 | 1.49(1.44-1.54) | <0.001 |

| | | | | | | | | |
|---|---|---|---|---|---|---|---|---|
| NLR quartiles | | | | | | | | |
| Q1(≤1.48) | Reference | | Reference | | Reference | | Reference | |
| Q2(1.48-1.88) | 1.40(1.29-1.53) | <0.001 | 1.41(1.29-1.54) | <0.001 | 1.37(1.25-1.50) | <0.001 | 1.36(1.24-1.49) | <0.001 |
| Q3(1.88-2.39) | 2.33(2.14-2.54) | <0.001 | 2.40(2.19-2.62) | <0.001 | 2.28(2.08-2.50) | <0.001 | 2.25(2.05-2.47) | <0.001 |
| Q4(>2.39) | 3.13(2.87-3.41) | <0.001 | 3.33(3.03-3.65) | <0.001 | 3.06(2.78-3.36) | <0.001 | 3.01(2.74-3.31) | <0.001 |
| *P* for trend | | <0.001 | | <0.001 | | <0.001 | | <0.001 |
| NPR per SD increase | 1.17(1.13-1.20) | <0.001 | 1.22(1.18-1.26) | <0.001 | 1.17(1.13-1.22) | <0.001 | 1.18(1.14-1.22) | <0.001 |
| NPR quartiles | | | | | | | | |
| Q1(≤0.011) | Reference | | Reference | | Reference | | Reference | |
| Q2(0.011-0.014) | 1.22(1.13-1.33) | <0.001 | 1.23(1.13-1.35) | <0.001 | 1.21(1.11-1.33) | <0.001 | 1.20(1.10-1.32) | <0.001 |
| Q3(0.014-0.018) | 1.28(1.18-1.39) | <0.001 | 1.35(1.23-1.47) | <0.001 | 1.27(1.16-1.39) | <0.001 | 1.26(1.15-1.38) | <0.001 |
| Q4(>0.018) | 1.59(1.46-1.72) | <0.001 | 1.78(1.62-1.95) | <0.001 | 1.63(1.48-1.79) | <0.001 | 1.62(1.48-1.79) | <0.001 |
| *P* for trend | | <0.001 | | <0.001 | | <0.001 | | <0.001 |
| PLR per SD increase | 1.25(1.21-1.29) | <0.001 | 1.26(1.22-1.31) | <0.001 | 1.25(1.21-1.30) | <0.001 | 1.24(1.20-1.29) | <0.001 |
| PLR quartiles | | | | | | | | |
| Q1(≤101.76) | Reference | | Reference | | Reference | | Reference | |
| Q2(101.76-125.56) | 1.21(1.11-1.31) | <0.001 | 1.18(1.08-1.29) | <0.001 | 1.16(1.06-1.27) | 0.001 | 1.15(1.05-1.26) | 0.002 |
| Q3(125.56-156.09) | 1.46(1.34-1.59) | <0.001 | 1.44(1.31-1.57) | <0.001 | 1.40(1.28-1.54) | <0.001 | 1.39(1.26-1.52) | <0.001 |
| Q4(>156.09) | 1.87(1.72-2.03) | <0.001 | 1.87(1.72-2.07) | <0.001 | 1.84(1.67-2.02) | <0.001 | 1.80(1.64-1.98) | <0.001 |
| *P* for trend | | <0.001 | | <0.001 | | <0.001 | | <0.001 |

Model 1: adjusted for age, sex, smoking, alcohol consumption, hypertension duration, BMI, CAD, dyslipidemia;

Model 2: adjusted for variables in Model 1: SBP, DBP, FBG, HbAlc, ALT, AST, TC, TG, HDL, LDL, Scr, UA, serum potassium, serum sodium, hs-CRP, TyG index.

Model 3: adjusted for variables in Model 2 plus ACEI/ARB, CCB, β-blocker, Diuretic, lipid-lowering agents, and antidiabetic agents.

Abbreviations: SD, standard deviation; OR, odds ratio; CI, confidence interval. Other abbreviations are as defined in Table 1.

**Table S6.** Sensitivity analysis of the association between inflammatory biomarkers and the Odds of LVH without adjustment for Hs-CRP.

| | Unadjusted | | Model 1 | | Model 2 | | Model 3 | |
|---|---|---|---|---|---|---|---|---|
| | OR (95%CI) | *P* value | OR (95%CI) | *P* value | OR (95%CI) | *P* value | OR (95%CI) | *P* value |
| AISI per SD increase | 1.40(1.36-1.45) | <0.001 | 1.41(1.36-1.45) | <0.001 | 1.40(1.35-1.45) | <0.001 | 1.39(1.34-1.44) | <0.001 |
| AISI quartiles | | | | | | | | |
| Q1(≤121.71) | Reference | | Reference | | Reference | | Reference | |
| Q2(121.71-187.12) | 1.40(1.30-1.52) | <0.001 | 1.38(1.27-1.50) | <0.001 | 1.39(1.27-1.51) | <0.001 | 1.37(1.26-1.49) | <0.001 |
| Q3(187.12-274.14) | 2.28(2.11-2.46) | <0.001 | 2.27(2.09-2.46) | <0.001 | 2.25(2.07-2.45) | <0.001 | 2.23(2.05-2.43) | <0.001 |
| Q4(>274.14) | 2.63(2.43-2.84) | <0.001 | 2.69(2.47-2.92) | <0.001 | 2.66(2.44-2.90) | <0.001 | 2.61(2.40-2.85) | <0.001 |
| *P* for trend | | <0.001 | | <0.001 | | <0.001 | | <0.001 |
| SIRI per SD increase | 1.35(1.31-1.39) | <0.001 | 1.38(1.34-1.43) | <0.001 | 1.35(1.31-1.40) | <0.001 | 1.34(1.30-1.40) | <0.001 |
| SIRI quartiles | | | | | | | | |
| Q1(≤0.54) | Reference | | Reference | | Reference | | Reference | |
| Q2(0.54-0.79) | 1.32(1.22-1.42) | <0.001 | 1.33(1.23-1.45) | <0.001 | 1.30(1.20-1.41) | <0.001 | 1.29(1.19-1.40) | <0.001 |
| Q3(0.79-1.10) | 1.98(1.84-2.14) | <0.001 | 2.07(1.90-2.25) | <0.001 | 2.01(1.85-2.19) | <0.001 | 1.99(1.82-2.16) | <0.001 |
| Q4(>1.10) | 2.38(2.20-2.57) | <0.001 | 2.57(2.36-2.80) | <0.001 | 2.44(2.24-2.66) | <0.001 | 2.40(2.20-2.62) | <0.001 |
| *P* for trend | | <0.001 | | <0.001 | | <0.001 | | <0.001 |
| SII per SD increase | 1.78(1.73-1.84) | <0.001 | 1.78(1.72-1.84) | <0.001 | 1.76(1.70-1.82) | <0.001 | 1.75(1.69-1.81) | <0.001 |
| SII quartiles | | | | | | | | |
| Q1(≤332.35) | Reference | | Reference | | Reference | | Reference | |
| Q2(332.35-445.82) | 1.38(1.27-1.49) | <0.001 | 1.37(1.26-1.49) | <0.001 | 1.36(1.25-1.50) | <0.001 | 1.35(1.24-1.47) | <0.001 |
| Q3(445.82-593.03) | 2.51(2.33-2.72) | <0.001 | 2.49(2.29-2.70) | <0.001 | 2.48(2.28-2.70) | <0.001 | 2.45(2.25-2.67) | <0.001 |
| Q4(>593.03) | 3.76(3.47-4.08) | <0.001 | 3.75(3.44-4.08) | <0.001 | 3.67(3.36-4.00) | <0.001 | 3.60(3.30-3.93) | <0.001 |
| *P* for trend | | <0.001 | | <0.001 | | <0.001 | | <0.001 |

| | | | | | | | | |
|---|---|---|---|---|---|---|---|---|
| NLR per SD increase | 1.51(1.46-1.55) | <0.001 | 1.54(1.49-1.59) | <0.001 | 1.49(1.44-1.54) | <0.001 | 1.48(1.43-1.53) | <0.001 |
| NLR quartiles | | | | | | | | |
| Q1(≤1.49) | Reference | | Reference | | Reference | | Reference | |
| Q2(1.49-1.89) | 1.39(1.29-1.50) | <0.001 | 1.40(1.29-1.52) | <0.001 | 1.37(1.26-1.49) | <0.001 | 1.36(1.25-1.48) | <0.001 |
| Q3(1.89-2.40) | 2.36(2.19-2.55) | <0.001 | 2.44(2.25-2.66) | <0.001 | 2.33(2.14-2.54) | <0.001 | 2.31(2.12-2.51) | <0.001 |
| Q4(>2.40) | 3.10(2.87-3.36) | <0.001 | 3.29(3.02-3.58) | <0.001 | 3.04(2.78-3.31) | <0.001 | 2.99(2.74-3.26) | <0.001 |
| *P* for trend | | <0.001 | | <0.001 | | <0.001 | | <0.001 |
| NPR per SD increase | 1.19(1.16-1.23) | <0.001 | 1.22(1.18-1.26) | <0.001 | 1.18(1.15-1.22) | <0.001 | 1.19(1.15-1.22) | <0.001 |
| NPR quartiles | | | | | | | | |
| Q1(≤0.011) | Reference | | Reference | | Reference | | Reference | |
| Q2(0.011-0.015) | 1.25(1.16-1.35) | <0.001 | 1.25(1.16-1.36) | <0.001 | 1.23(1.14-1.34) | <0.001 | 1.23(1.13-1.33) | <0.001 |
| Q3(0.015-0.018) | 1.37(1.27-1.48) | <0.001 | 1.39(1.28-1.51) | <0.001 | 1.32(1.21-1.44) | <0.001 | 1.31(1.21-1.43) | <0.001 |
| Q4(>0.018) | 1.70(1.58-1.84) | <0.001 | 1.82(1.67-1.98) | <0.001 | 1.68(1.54-1.83) | <0.001 | 1.68(1.54-1.84) | <0.001 |
| *P* for trend | | <0.001 | | <0.001 | | <0.001 | | <0.001 |
| PLR per SD increase | 1.23(1.20-1.27) | <0.001 | 1.26(1.23-1.30) | <0.001 | 1.25(1.22-1.30) | <0.001 | 1.25(1.21-1.29) | <0.001 |
| PLR quartiles | | | | | | | | |
| Q1(≤100.06) | Reference | | Reference | | Reference | | Reference | |
| Q2(100.06-124.48) | 1.22(1.13-1.32) | <0.001 | 1.23(1.14-1.34) | <0.001 | 1.22(1.12-1.37) | 0.001 | 1.21(1.12-1.32) | 0.002 |
| Q3(124.48-154.79) | 1.42(1.32-1.53) | <0.001 | 1.46(1.34-1.58) | <0.001 | 1.44(1.32-1.56) | <0.001 | 1.39(1.30-1.54) | <0.001 |
| Q4(>154.79) | 1.85(1.71-1.99) | <0.001 | 1.94(1.79-2.11) | <0.001 | 1.92(1.76-2.09) | <0.001 | 1.88(1.72-2.05) | <0.001 |
| *P* for trend | | <0.001 | | <0.001 | | <0.001 | | <0.001 |

Model 1: adjusted for age, sex, smoking, alcohol consumption, hypertension duration, BMI, CAD, dyslipidemia;

Model 2: adjusted for variables in Model 1: SBP, DBP, FBG, HbAlc, ALT, AST, TC, TG, HDL, LDL, Scr, UA, serum potassium, serum sodium, TyG index.

Model 3: adjusted for variables in Model 2 plus ACEI/ARB, CCB, β-blocker, Diuretic, lipid-lowering agents, and antidiabetic agents.

Abbreviations: SD, standard deviation; OR, odds ratio; CI, confidence interval. Other abbreviations are as defined in Table 1.

**Table S7.** Sensitivity analysis of the associations between inflammatory indices and LVH across different recruitment periods.

| Inflammatory indices | 2011–2015 OR (95% CI) (n = 4542) | P value | 2016–2019 OR (95% CI) (n = 9031) | P value | 2020–2023 OR (95% CI) (n = 7913) | P value | P for interaction |
|---|---|---|---|---|---|---|---|
| AISI, per SD increase | 1.93 (1.76-2.13) | <0.001 | 1.36 (1.29-1.43) | <0.001 | 1.40 (1.32-1.48) | <0.001 | <0.001 |
| SIRI, per SD increase | 1.71 (1.56-1.87) | <0.001 | 1.31 (1.25-1.38) | <0.001 | 1.41 (1.33-1.49) | <0.001 | <0.001 |
| SII, per SD increase | 1.92 (1.77-2.09) | <0.001 | 1.78 (1.68-1.88) | <0.001 | 1.72 (1.63-1.82) | <0.001 | 0.065 |
| NLR, per SD increase | 1.50 (1.39-1.62) | <0.001 | 1.48 (1.40-1.56) | <0.001 | 1.48 (1.41-1.56) | <0.001 | 0.863 |
| NPR, per SD increase | 1.16 (1.09-1.23) | <0.001 | 1.14 (1.08-1.20) | <0.001 | 1.20 (1.14-1.27) | <0.001 | 0.573 |
| PLR, per SD increase | 1.29 (1.20-1.40) | <0.001 | 1.28 (1.21-1.34) | <0.001 | 1.25 (1.19-1.32) | <0.001 | 0.68 |

Separate multivariable logistic regression analyses were performed for the recruitment periods 20112015, 2016-2019, and 2020-2023. Each inflammatory index was entered individually into the fully adjusted model using the same covariate set as Model 3 of the primary analysis. Model 3: adjusted for age, sex, smoking, alcohol consumption, hypertension duration, BMI, CAD, diabetes mellitus, dyslipidemia, SBP, DBP, FBG, HbAlc, ALT, AST, TC, TG, HDL, LDL, Scr, UA, serum potassium, serum sodium, hs-CRP, TyG index, ACEI/ARB, CCB, β-blocker, Diuretic, lipid-lowering drugs, Antidiabetic agents. Abbreviations: SD, standard deviation; OR, odds ratio; CI, confidence interval.

**Table S8.** Interaction estimates for seven subgroups in the associations between inflammatory indices and LVH.

| Inflammatory Indices | Subgroup | Interaction-term OR (95% CI) | P for interaction |
|---|---|---|---|
| AISI | Age (≥60 vs. <60 years) | 1.06 (0.97-1.15) | 0.219 |
| | Sex (Male vs. Female) | 0.94 (0.88-1.00) | 0.066 |
| | BMI (≥24 vs. <24 kg/m²) | 0.94 (0.87-1.02) | 0.139 |
| | Smoking (Yes vs. No) | 0.93 (0.87-0.99) | 0.029 |
| | Alcohol consumption (Yes vs. No) | 0.99 (0.93-1.06) | 0.784 |
| | Hypertension Duration (≥5 vs. <5 years) | 1.08 (1.01-1.16) | 0.018 |
| | Dyslipidemia (Yes vs. No) | 0.95 (0.89-1.02) | 0.147 |
| SIRI | Age (≥60 vs. <60 years) | 0.93 (0.86-1.00) | 0.064 |
| | Sex (Male vs. Female) | 0.93 (0.87-1.00) | 0.039 |
| | BMI (≥24 vs. <24 kg/m²) | 1.00 (0.92-1.08) | 0.917 |
| | Smoking (Yes vs. No) | 0.92 (0.87-0.99) | 0.016 |
| | Alcohol consumption (Yes vs. No) | 0.98 (0.92-1.05) | 0.629 |
| | Hypertension Duration (≥5 vs. <5 years) | 1.04 (0.97-1.10) | 0.266 |
| | Dyslipidemia (Yes vs. No) | 1.02 (0.96-1.09) | 0.466 |
| SII | Age (≥60 vs. <60 years) | 0.98 (0.90-1.06) | 0.602 |
| | Sex (Male vs. Female) | 1.10 (1.03-1.17) | 0.007 |
| | BMI (≥24 vs. <24 kg/m²) | 1.03 (0.95-1.11) | 0.481 |
| | Smoking (Yes vs. No) | 1.08 (1.01-1.16) | 0.03 |
| | Alcohol consumption (Yes vs. No) | 1.09 (1.02-1.18) | 0.016 |
| | Hypertension Duration (≥5 vs. <5 years) | 1.04 (0.97-1.11) | 0.275 |
| | Dyslipidemia (Yes vs. No) | 1.04 (0.97-1.12) | 0.237 |
| NLR | Age (≥60 vs. <60 years) | 0.87 (0.80-0.94) | <0.001 |
| | Sex (Male vs. Female) | 1.08 (1.01-1.15) | 0.023 |
| | BMI (≥24 vs. <24 kg/m²) | 1.04 (0.96-1.12) | 0.324 |
| | Smoking (Yes vs. No) | 1.05 (0.98-1.12) | 0.159 |
| | Alcohol consumption (Yes vs. No) | 1.06 (0.99-1.13) | 0.121 |
| | Hypertension Duration (≥5 vs. <5 years) | 0.96 (0.90-1.02) | 0.208 |
| | Dyslipidemia (Yes vs. No) | 1.04 (0.98-1.11) | 0.233 |
| NPR | Age (≥60 vs. <60 years) | 0.90 (0.84-0.97) | 0.006 |
| | Sex (Male vs. Female) | 1.05 (0.98-1.12) | 0.15 |
| | BMI (≥24 vs. <24 kg/m²) | 0.99 (0.93-1.07) | 0.876 |
| | Smoking (Yes vs. No) | 0.99 (0.93-1.05) | 0.756 |
| | Alcohol consumption (Yes vs. No) | 1.01 (0.95-1.07) | 0.783 |
| | Hypertension Duration (≥5 vs. <5 years) | 0.97 (0.91-1.03) | 0.269 |
| | Dyslipidemia (Yes vs. No) | 0.96 (0.91-1.02) | 0.236 |
| PLR | Age (≥60 vs. <60 years) | 0.99 (0.92-1.06) | 0.72 |
| | Sex (Male vs. Female) | 1.11 (1.05-1.18) | <0.001 |
| | BMI (≥24 vs. <24 kg/m²) | 1.04 (0.97-1.11) | 0.26 |
| | Smoking (Yes vs. No) | 1.14 (1.07-1.22) | <0.001 |
| | Alcohol consumption (Yes vs. No) | 1.08 (1.01-1.16) | 0.017 |
| | Hypertension Duration (≥5 vs. <5 years) | 0.99 (0.93-1.05) | 0.789 |
| | Dyslipidemia (Yes vs. No) | 1.06 (1.00-1.12) | 0.057 |

Abbreviations: OR, odds ratio; Cl, confidence interval; Interaction-term ORs were obtained from multivariable logistic regression models containing the multiplicative interaction term between each inflammatory index and the corresponding subgroup variable.

**Table S9**. Diagnostic performance of individual inflammatory biomarkers for LVH

| Index | AUC | 95%CI_Low | 95%CI_High | Specificity | Sensitivity | PPV | NPV |
|---|---|---|---|---|---|---|---|
| AISI | 0.607 | 0.6 | 0.615 | 0.58 | 0.601 | 0.608 | 0.571 |
| SIRI | 0.593 | 0.586 | 0.601 | 0.568 | 0.592 | 0.598 | 0.561 |
| SII | 0.656 | 0.649 | 0.663 | 0.62 | 0.617 | 0.639 | 0.598 |
| NLR | 0.623 | 0.615 | 0.63 | 0.559 | 0.646 | 0.614 | 0.592 |
| NPR | 0.553 | 0.545 | 0.561 | 0.536 | 0.543 | 0.56 | 0.519 |
| PLR | 0.564 | 0.556 | 0.571 | 0.519 | 0.577 | 0.566 | 0.53 |

Abbreviations: AUC, area under the curve; Positive-pv, positive predictive value; Negative-pv, negative predictive value. Other abbreviations are as defined in Table 1.

**Table S10.** Bootstrap optimism-corrected internal validation of the predictive models for LVH.

| Model | Apparent_AUC | Optimism_Penalty | Corrected_AUC | Corrected_Slope | Corrected_Brier |
|---|---|---|---|---|---|
| Baseline Model | 0.7142 | 0.0003 | 0.7138 | 0.9979 | 0.215 |
| Baseline + AISI | 0.7281 | 0.0002 | 0.7279 | 0.9987 | 0.2107 |
| Baseline + SIRI | 0.7262 | 0.0003 | 0.7258 | 0.9981 | 0.2112 |
| Baseline + SII | 0.7504 | 0.0003 | 0.7501 | 0.9984 | 0.2029 |
| Baseline + NLR | 0.7366 | 0.0003 | 0.7363 | 0.9984 | 0.2078 |
| Baseline + NPR | 0.719 | 0.0005 | 0.7185 | 0.9971 | 0.2135 |
| Baseline + PLR | 0.7206 | 0.0003 | 0.7203 | 0.9984 | 0.213 |

Abbreviations: AUC, area under the curve. Other abbreviations are as defined in Table 1.

STROBE Statement—checklist of items that should be included in reports of observational studies

| | Item No | Recommendation | Page No |
|---|---|---|---|
| **Title and abstract** | 1 | (*a*) Indicate the study's design with a commonly used term in the title or the abstract | 1 |
| | | (*b*) Provide in the abstract an informative and balanced summary of what was done and what was found | 1 |
| **Introduction** | | | |
| Background/rationale | 2 | Explain the scientific background and rationale for the investigation being reported | 2 |
| Objectives | 3 | State specific objectives, including any prespecified hypotheses | 3 |
| **Methods** | | | |
| Study design | 4 | Present key elements of study design early in the paper | 3 |
| Setting | 5 | Describe the setting, locations, and relevant dates, including periods of recruitment, exposure, follow-up, and data collection | 3 |
| Participants | 6 | (*a*) *Cohort study*—Give the eligibility criteria, and the sources and methods of selection of participants. Describe methods of follow-up<br>*Case–control study*—Give the eligibility criteria, and the sources and methods of case ascertainment and control selection. Give the rationale for the choice of cases and controls<br>*Cross-sectional study*—Give the eligibility criteria, and the sources and methods of selection of participants | 3 |
| | | (*b*) *Cohort study*—For matched studies, give matching criteria and number of exposed and unexposed<br>*Case–control study*—For matched studies, give matching criteria and the number of controls per case | |
| Variables | 7 | Clearly define all outcomes, exposures, predictors, potential confounders, and effect modifiers. Give diagnostic criteria, if applicable | 4 |
| Data sources/ measurement | 8* | For each variable of interest, give sources of data and details of methods of assessment (measurement). Describe comparability of assessment methods if there is more than one group | *4* |
| Bias | 9 | Describe any efforts to address potential sources of bias | 4 |
| Study size | 10 | Explain how the study size was arrived at | 4 |

| | | | |
|---|---|---|---|
| Quantitative variables | 11 | Explain how quantitative variables were handled in the analyses. If applicable, describe which groupings were chosen and why | 5 |
| Statistical methods | 12 | (*a*) Describe all statistical methods, including those used to control for confounding | 5 |
| | | (*b*) Describe any methods used to examine subgroups and interactions | 5 |
| | | (*c*) Explain how missing data were addressed | 5 |
| | | (*d*) *Cohort study*—If applicable, explain how loss to follow-up was addressed<br>*Case–control study*—If applicable, explain how matching of cases and controls was addressed<br>*Cross-sectional study*—If applicable, describe analytical methods taking account of sampling strategy | 5 |
| | | (*e*) Describe any sensitivity analyses | |

**Results**

| | | | |
|---|---|---|---|
| Participants | 13* | (a) Report numbers of individuals at each stage of study—eg numbers potentially eligible, examined for eligibility, confirmed eligible, included in the study, completing follow-up, and analyzed | 3 |
| | | (b) Give reasons for non-participation at each stage | 3 |
| | | (c) Consider use of a flow diagram | 3 |
| Descriptive data | 14* | (a) Give characteristics of study participants (eg demographic, clinical, social) and information on exposures and potential confounders | 5 |
| | | (b) Indicate number of participants with missing data for each variable of interest | 5 |
| | | (c) *Cohort study*—Summarize follow-up time (e.g., average and total amount) | 5 |
| Outcome data | 15* | *Cohort study*—Report numbers of outcome events or summary measures over time | |
| | | *Case–control study*—Report numbers in each exposure category, or summary measures of exposure | |
| | | *Cross-sectional study*—Report numbers of outcome events or summary measures | *5* |
| Main results | 16 | (*a*) Give unadjusted estimates and, if applicable, confounder-adjusted estimates and their precision (e.g., 95% confidence interval). Make clear which confounders were adjusted for and why they were included | 5 |
| | | (*b*) Report category boundaries when continuous variables were categorized | 5 |
| | | (*c*) If relevant, consider translating estimates of relative risk into absolute risk for a meaningful time period | 5 |

| | | | |
|---|---|---|---|
| Other analyses | 17 | Report other analyses done—eg analyses of subgroups and interactions, and sensitivity analyses | 6-15 |
| **Discussion** | | | |
| Key results | 18 | Summarize key results with reference to study objectives | 15 |
| Limitations | 19 | Discuss limitations of the study, taking into account sources of potential bias or imprecision. Discuss both direction and magnitude of any potential bias | 17 |
| Interpretation | 20 | Give a cautious overall interpretation of results considering objectives, limitations, multiplicity of analyses, results from similar studies, and other relevant evidence | 17 |
| Generalisability | 21 | Discuss the generalisability (external validity) of the study results | 17 |
| **Other information** | | | |
| Funding | 22 | Give the source of funding and the role of the funders for the present study and, if applicable, for the original study on which the present article is based | 19 |

## S3.Supplementary Figures

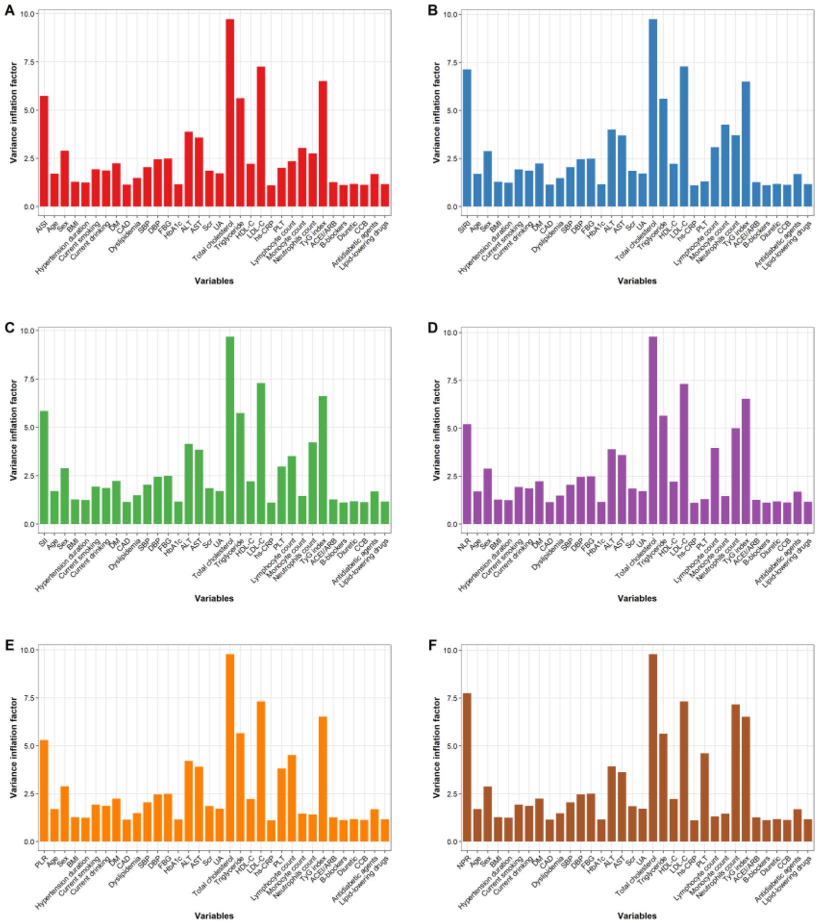


**Figure S1.** Collinearity diagnostics step.

VIF = 1/(1-R2). VIF step-by-step screening method: Calculate the VIF of each variable. To avoid redundancy of information among lipid-related variables and instability in model estimates, we retained LDL-C and excluded TC from the final multivariate model.

Abbreviations: VIF: variance inflation factor. Other abbreviations are as defined in Table 1.

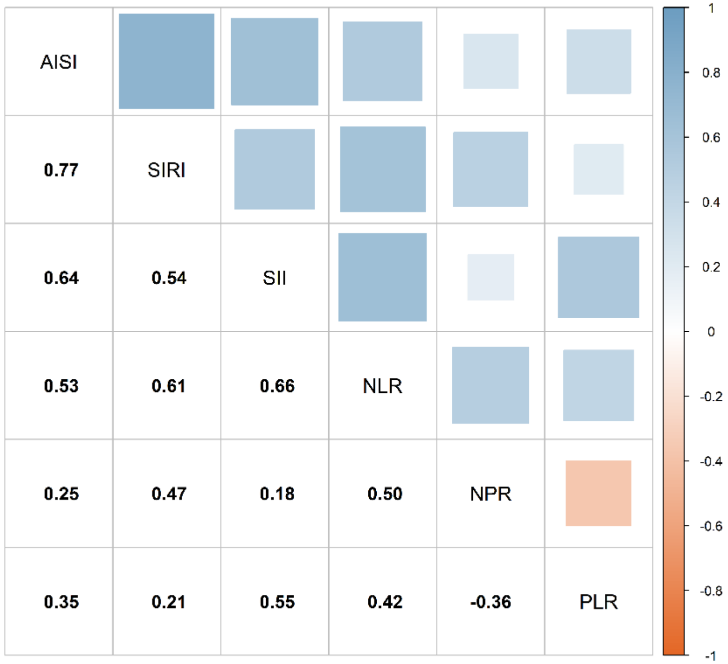


**Figure S2.** Spearman correlation matrix among the six inflammatory indices.

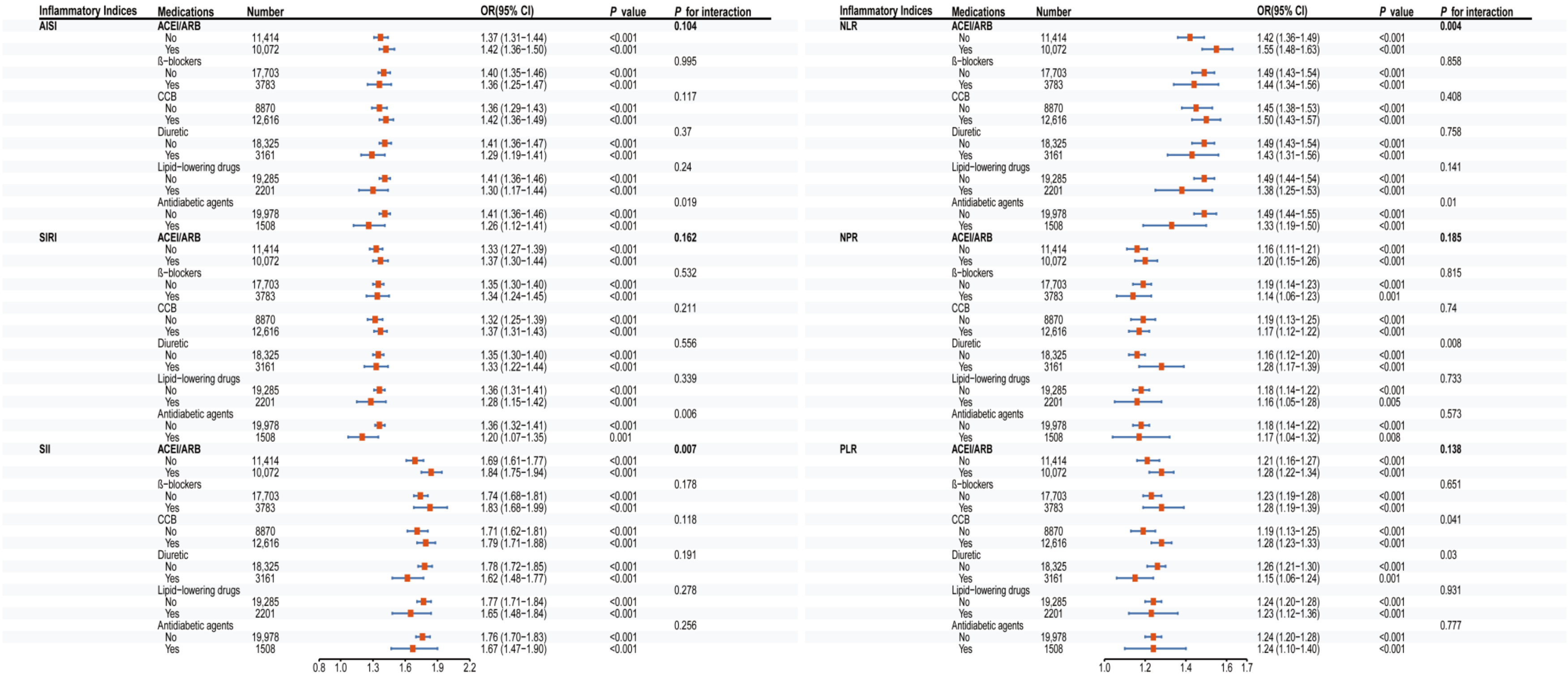


**Figure S3.** Association of inflammatory biomarkers with LVH stratified by medication use.

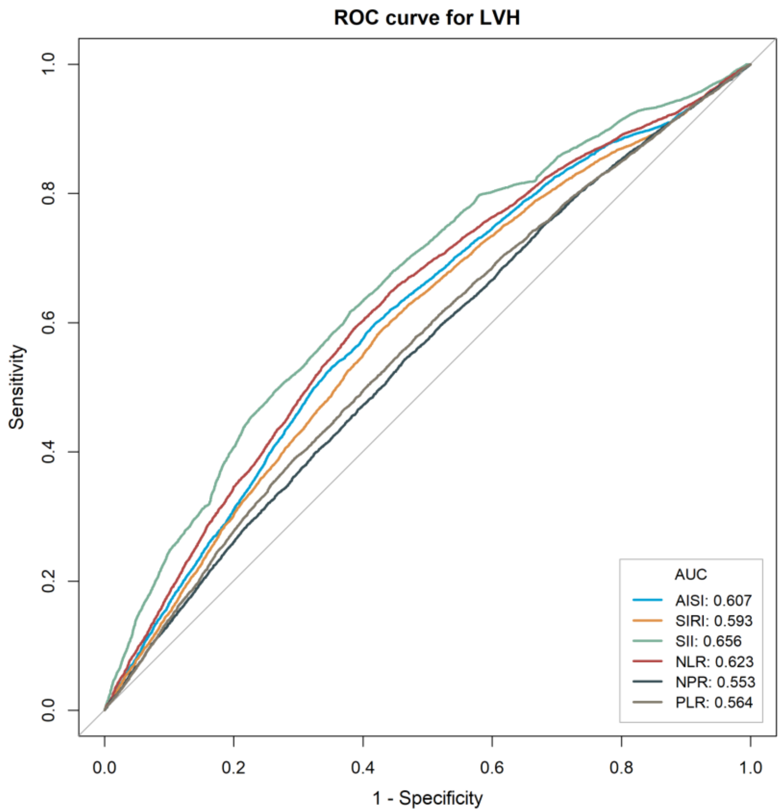


**Figure S4.** Receiver operating characteristic (ROC) curve analysis of individual inflammatory biomarkers for LVH.

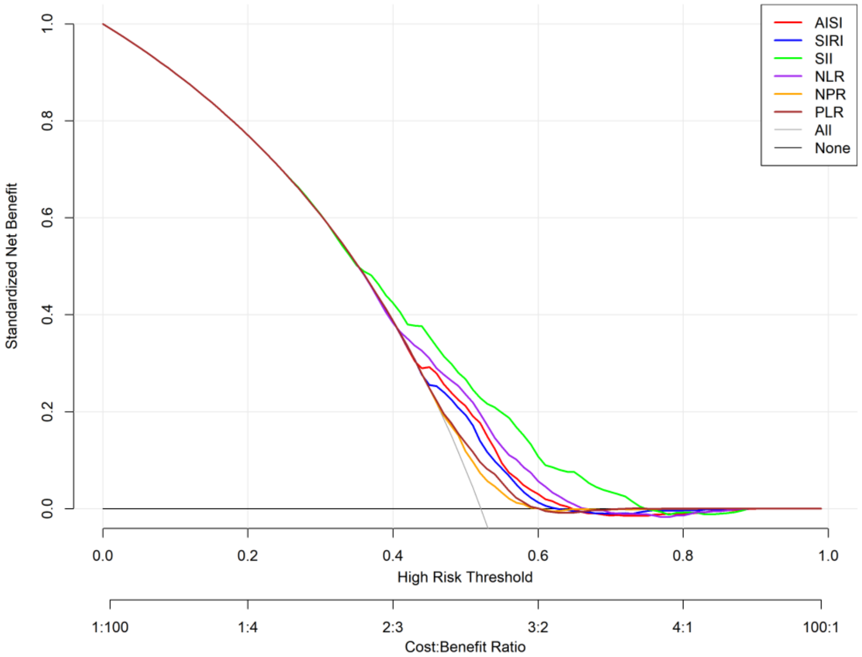


**Figure S5.** Decision curve analysis of individual inflammatory biomarkers for the identification of LVH.

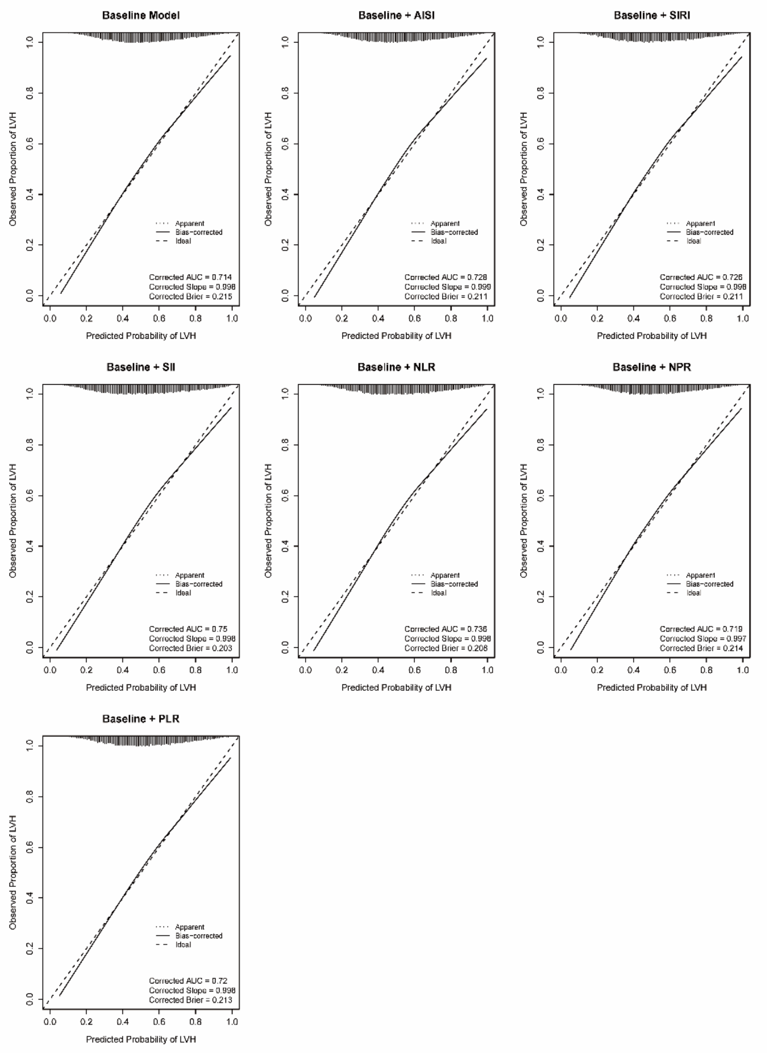


**Figure S6.** Calibration curves of the predictive models for LVH after bootstrap optimism correction.
